# SIRT7 promotes lung cancer progression by destabilizing the tumor suppressor ARF

**DOI:** 10.1073/pnas.2409269121

**Published:** 2024-06-13

**Authors:** Poonam Kumari, Shahriar Tarighi, Eva Fuchshuber, Luhan Li, Irene Fernández-Duran, Meilin Wang, Joshua Ayoson, Jose Manuel Castelló-García, Andrés Gámez-García, Maria Espinosa-Alcantud, Krishnamoorthy Sreenivasan, Stefan Guenther, Mireia Olivella, Rajkumar Savai, Shijing Yue, Alejandro Vaquero, Thomas Braun, Alessandro Ianni

**Affiliations:** ^a^Department of Cardiac Development and Remodeling, Max-Planck-Institute for Heart and Lung Research, Bad Nauheim 61231, Germany; ^b^School of Medicine, Nankai University, Tianjin 300071, China; ^c^Chromatin Biology Laboratory, Josep Carreras Leukaemia Research Institute, Badalona, Barcelona, Catalonia 08916, Spain; ^d^Department of Lung Development and Remodeling, Max-Planck-Institute for Heart and Lung Research, Bad Nauheim 61231, Germany; ^e^Facultat de Ciències, Tecnologia I Enginyeries, Universitat de Vic-Universitat Central de Catalunya, Vic, Barcelona 08500, Spain; ^f^Institut de Recerca i Innovació en Ciències de la Vida i de la Salut a la Catalunya Central, Vic, Barcelona 08500, Spain; ^g^Lung Microenvironmental Niche in Cancerogenesis, Institute for Lung Health, Justus Liebig University, Giessen D-35392, Germany

**Keywords:** Sirtuins, SIRT7, ARF, nucleophosmin, lung cancer

## Abstract

Sirtuin 7 (SIRT7) is a member of the mammalian family of nicotinamide adenine dinucleotide (NAD^+^)-dependent histone/protein deacetylases, known as sirtuins. It acts as a potent oncogene in numerous malignancies, but the molecular mechanisms employed by SIRT7 to sustain lung cancer progression remain largely uncharacterized. We demonstrate that SIRT7 exerts oncogenic functions in lung cancer cells by destabilizing the tumor suppressor alternative reading frame (ARF). SIRT7 directly interacts with ARF and prevents binding of ARF to nucleophosmin, thereby promoting proteasomal-dependent degradation of ARF. We show that SIRT7-mediated degradation of ARF increases expression of protumorigenic genes and stimulates proliferation of non-small-cell lung cancer (NSCLC) cells both in vitro and in vivo in a mouse xenograft model. Bioinformatics analysis of transcriptome data from human lung adenocarcinomas revealed a correlation between *SIRT7* expression and increased activity of genes normally repressed by ARF. We propose that disruption of SIRT7–ARF signaling stabilizes ARF and thus attenuates cancer cell proliferation, offering a strategy to mitigate NSCLC progression.

## Significance

We found that the NAD^+^-dependent deacetylase SIRT7 directly interacts and destabilizes the tumor suppressor ARF. Mechanistically, SIRT7 prevents association of ARF to Nucleophosmin and thereby facilitates ARF proteasomal-dependent degradation in lung cancer cells. The study unveils a unique mechanism by which SIRT7 promotes proliferation of non-small-cell lung cancer that may be exploited to increase cellular levels of ARF for antitumor therapies in cancers with intact ARF expression.

The ARF tumor suppressor (p14ARF in humans and p19ARF in mice) was originally identified as an alternative transcript of the INK4b-ARF-INK4a (*CDKN2A*) locus located on human chromosome 9p21. The locus also codes for two additional cyclin-dependent kinase inhibitors (p15^INK4b^ and p16^INK4a^) and is deleted in a broad range of tumors such as glioblastoma, lung and bladder cancer among others (1). Inactivation of *ARF* accelerates tumorigenesis, which is further enhanced by deletion of both *ARF* and *p16 ^INK4A^* (1). Mice lacking the first exon 1β, specific for *ARF*, are highly tumor-prone and die of cancers within 15 mo of age (2). Similar effects occur when exon 2 of *INK4a*/*ARF* is deleted with is shared with *p16 ^INK4A^* (3). ARF acts as a potent tumor suppressor by controlling different molecular pathways, including stabilization of the tumor suppressor p53 by inhibition of the ubiquitin ligase MDM2. Consequently, ARF promotes cell cycle arrest, apoptosis, and cellular senescence in a p53-dependent manner (4). However, ARF also inhibits cell proliferation in p53-deficient cells, indicating that ARF suppresses tumors by different mechanisms (4). Such mechanisms encompass inhibition of prominent oncogenes such as Myc and E2F1 (5), inhibition of ribosome biogenesis (6, 7), induction of autophagy (8), and destabilization of transcription factors involved in cell cycle progression (9). In addition, ARF promotes the recruitment of histone deacetylase 1 (HDAC1) to target genes involved in cell proliferation and survival such as cyclin E1 (*CCNE1*), enabling epigenetic silencing by facilitating HDAC1-dependent deacetylation of histone 2B at lysine 20 (H2BK20) (10).

*ARF* gene expression is controlled by stress and a broad range of oncogenes and oncogene-activated factors such as Ras, c-Myc, E1A, and E2F1 that are directly recruited to the *ARF* promoter to stimulate transcription. Induction of ARF in response to oncogenes and stress counteracts malignant transformation by inducing cell cycle arrest, cellular senescence, and apoptosis but also by activating DNA repair (4, 11, 12). However, a subset of molecules rather exert oncogenic functions by reducing ARF levels (13, 14). ARF protein activity is also controlled by retention in the nucleolus (15) via interactions with Nucleophosmin (NPM) and Nucleostemin (15–17). The interaction of ARF with NPM prevents association with critical ubiquitin ligases, residing in the nucleoplasm, (17, 18). Disruption of NPM–ARF interactions by mutations or posttranslational modifications of NPM rapidly induce ARF degradation promoting tumorigenesis (19–22).

Mammalian sirtuins comprise a family of enzymes that have been implicated in numerous biological functions such as cell proliferation, apoptosis, metabolic control, DNA repair, and others. Sirtuins primarily act as NAD^+^-dependent protein deacetylases, although some members also display mono-ADP ribosylation activity as well as other less-characterized activities (23, 24). SIRT7 is the only member of the family that is primarily enriched in the nucleolus where it contributes to different nucleolar functions such as rDNA transcription, pre-rRNA processing, and maintenance of rDNA stability (25). SIRT7 is up-regulated in numerous human cancers, stimulating cell cycle progression, survival of cancer cells, and metastasis (26). We recently demonstrated that SIRT7 is a critical regulator of NPM functions in the nucleolus following genotoxic stress by promoting its translocation into the nucleoplasm via deacetylation following ultraviolet-induced genotoxic stress. This process is crucial to enable NPM-mediated inhibition of MDM2, thus promoting p53 stabilization (27).

Here, we demonstrate that SIRT7 destabilizes ARF by disrupting its interaction with NPM through direct binding of SIRT7 to ARF. Destabilization of ARF by SIRT7 promotes proliferation of lung cancer cells both in vitro and in vivo. We propose that pharmacological manipulation of the SIRT7/NPM/ARF axis may be exploited for antitumor therapies in cancers with intact ARF expression.

## Results

### Depletion of *SIRT7* Increases ARF Levels in the Epithelial Cells of the Lung.

**Fig. 1.**
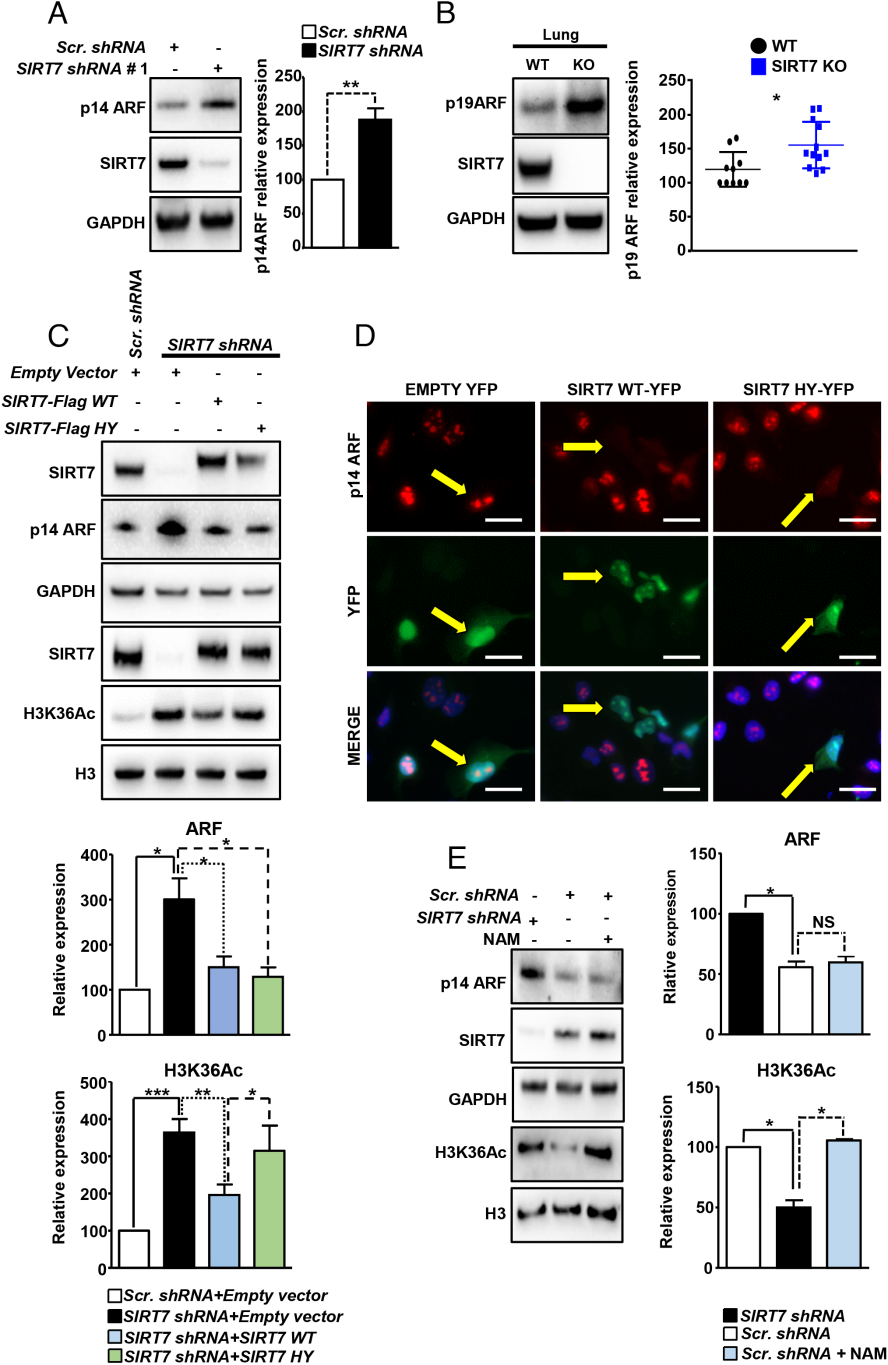
SIRT7 reduces ARF protein levels independently of its catalytic activity. (*A*) Western blot analysis of p14ARF levels in scrambled (*Scr. shRNA*) and *SIRT7* Knockdown (KD; *SIRT7 shRNA#*1) H1299 lung cancer cell lines. Quantification of ARF relative expression ± SD is shown on the *Right* (n = 6). (*B*) Western blot analysis of p19ARF levels in age-matched WT and *SIRT7* KO lungs. Quantification of p19ARF expression is shown in the histogram on the right (n = 10 WT and 12 KO). (*C*) Western blot analysis of p14ARF expression in *SIRT7* KD (*SIRT7 shRNA)* H1299 cells, transiently transfected with wild-type (WT) or catalytic inactive mutant (HY) SIRT7. Vectors were titrated to obtain physiological levels of SIRT7. H3K36Ac, a target of SIRT7 catalytic activity was used as a control. Quantification of ARF and H3K36Ac relative expression ± SD normalized on GAPDH and total histone 3 (H3), respectively, is shown in the histograms below (n = 7). (*D*) Immunofluorescence (IF) staining for p14ARF (red) and yellow fluorescent protein (YFP) in H1299 cells transiently transfected with empty vector (Empty YFP), YFP-tagged WT, and catalytic inactive mutant (HY) SIRT7. Cell nuclei were counterstained with 4′,6-Diamidino-2-Phenylindole (DAPI; n = 3; Scale bar: 20 µm). (*E*) Western blot analysis of p14ARF levels in *SIRT7* KD (*SIRT7 shRNA*) and scrambled (*Scr. shRNA*) H1299 treated with NAM (5 mM) for 24 h as indicated. GAPDH and total histone 3 (H3) were used as loading controls. Quantifications of relative p14ARF levels and H3K36Ac (normalized on GAPDH and total H3, respectively) ± SD are shown in the histograms on the *Right* (n = 3).

Since SIRT7 localizes in the nucleolus and interacts with NPM (27), a prominent regulator of ARF stability, we reasoned that SIRT7 may influence ARF protein levels in NSCLC (non-small-cell lung cancer) cells. We observed a significant increase of p14ARF protein levels in H1299 human lung cancer cells, in which *SIRT7* expression was suppressed by two independent *SIRT7*-targeting shRNAs compared to scrambled *shRNA* controls (Fig. 1*A* and *SI Appendix*, Fig. S1*A*). Similar results were obtained in both Calu-3 and PC-14 lung cancer cell lines following *shRNA*-mediated inhibition of *SIRT7* (*SI Appendix*, Fig. S1 *B* and *C*). Additionally, bioinformatics analyses revealed an inverse correlation between SIRT7 and ARF protein levels across diverse lung cancer cells (*SI Appendix*, Fig. S1*D*). Analysis of p19ARF expression in lung samples unveiled increased levels of ARF protein in *SIRT7* KO compared to WT (wild-type) mice, further emphasizing the reduced presence of ARF when SIRT7 is high (Fig. 1*B*).

To investigate whether the catalytic activity of SIRT7 is required to control ARF levels, we introduced WT or catalytic inactive mutant (HY) SIRT7 in *SIRT7* knockdown (KD) H1299 cell lines, taking care that exogenously introduced genes are expressed at physiological levels (Fig. 1*C*). Notably, Western blot analysis of p14ARF expression revealed a similar reduction of p14ARF levels in both WT and HY mutant SIRT7-expressing H1299 cells, indicating that SIRT7 deacetylation activity is not required for SIRT7-mediated inhibition of ARF (Fig. 1*C*). Additionally, immunofluorescence (IF) analysis revealed that expression of YFP-tagged SIRT7 WT and HY mutant SIRT7 dramatically reduces p14ARF in the nucleolus (Fig. 1*D*). Finally, inhibition of SIRT7 catalytic activity by the pan-sirtuin inhibitor nicotinamide (NAM) did not influence the levels of ARF in lung cancer cells. The dramatic increase of acetylated lysine 36 of histone 3 (H3K36Ac) levels, a prominent target of SIRT7 deacetylation activity (26), served as a control to confirm that the enzymatic activity of SIRT7 was successfully inhibited by NAM (Fig. 1*E*).

### SIRT7 Destabilizes ARF Protein by Promoting Ubiquitination and Proteasomal-Dependent Degradation.

**Fig. 2.**
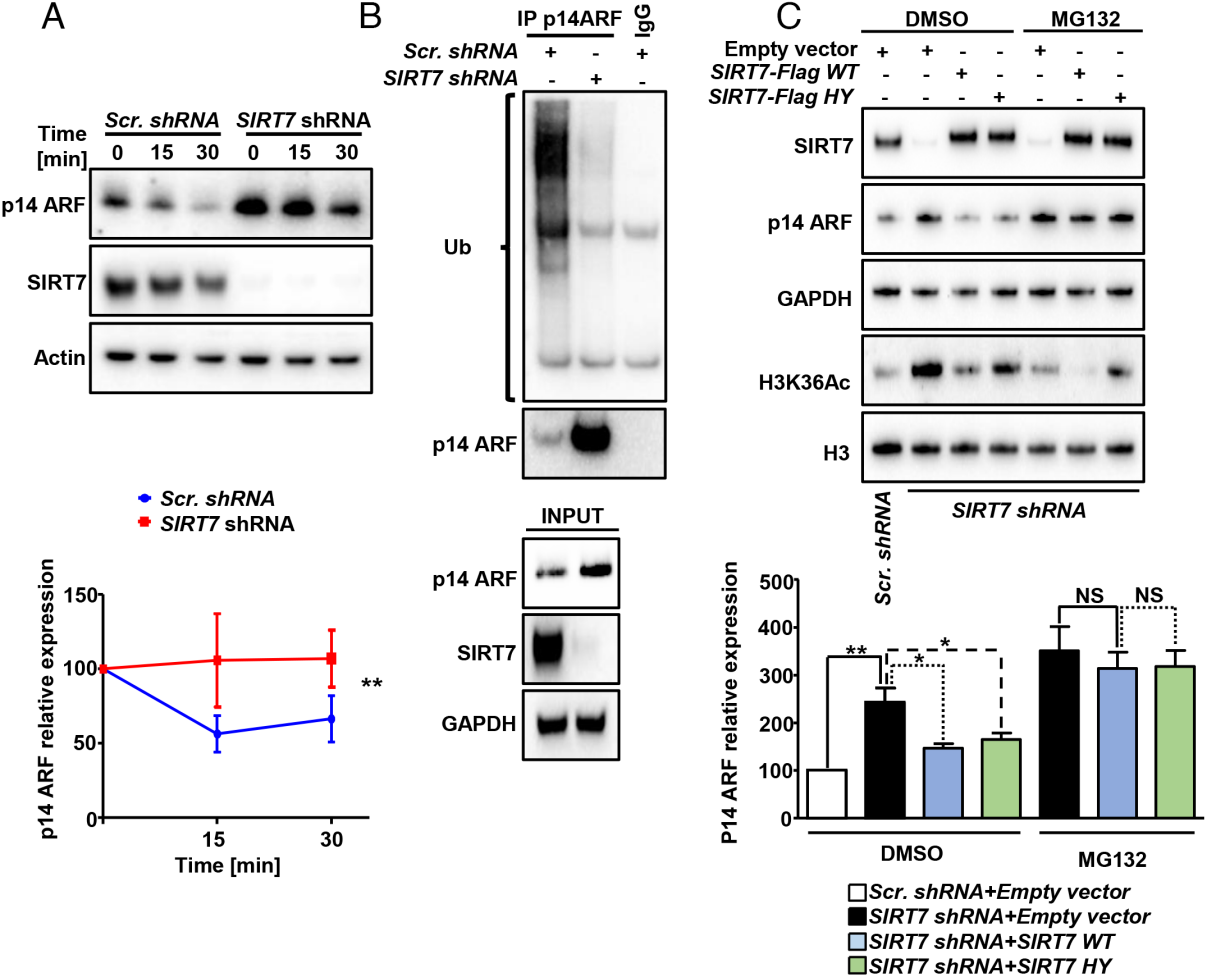
SIRT7 promotes ubiquitination and proteasomal-dependent degradation of ARF. (*A*) Western blot analysis of p14ARF levels in control (scrambled; *Scr. shRNA*) and *SIRT7* KD (*SIRT7 shRNA*) H1299 cells at indicated time points after treatment with CHX (50 µg/mL). Quantifications of p14ARF levels are given in the graph below (n = 4; two-way Anova statistic test). (*B*) Coupled immunoprecipitation (IP; anti-p14ARF antibody) and Western blot analysis (anti-ubiquitin antibody) of control (scrambled; *Scr. shRNA*) and *SIRT7* KD (*SIRT7 shRNA*) H1299 cells. A representative blot out of three independent experiments is shown (*Upper*). The membrane was reprobed with anti-p14ARF antibody (*Lower*). (*C*) Western blot analysis of p14ARF levels in scrambled and *SIRT7* KD cells transiently transfected with Flag-tagged WT and catalytic inactive (HY) SIRT7 as indicated, 5 h after treatment with 10 µM MG-132. DMSO was used as vehicle. Quantification of ARF levels ± SD is shown in the histograms below (n = 5).

To analyze whether SIRT7 controls ARF protein stability, we monitored p14ARF protein levels at different time points after treatment with the translation inhibitor cycloheximide (CHX) both in H1299 (Fig. 2*A*) and in Calu-3 (*SI Appendix*, Fig. S1*E*) lung cancer cell lines. *SIRT7*-depleted cells displayed higher stability of ARF protein compared to control cells, arguing for a posttranslational control mechanism. Next, we assessed the degree of ARF ubiquitination by Western blot analysis using an anti-ubiquitin antibody, which showed a dramatic reduction of ARF ubiquitination upon *SIRT7* downregulation in both H1299 and Calu-3 cells (Fig. 2*B* and *SI Appendix*, Fig. S1*F*, respectively). Furthermore, administration of the proteasome inhibitor MG132 restored ARF levels in *SIRT7*-depleted H1299 lung cancer cells transfected with WT and catalytic inactive mutant SIRT7 (Fig. 2*C*). MG132 treatment also normalized the unbalanced ARF levels in *SIRT7*-depleted H1299 cells (*SI Appendix*, Fig. S1*G*) although we still noted a slight, yet not significant increase in ARF levels in *SIRT7*-deficient H1299 cells after MG132 treatment, suggesting that additional mechanisms may be employed by SIRT7 to control ARF expression. RT-qPCR analysis of *ARF* expression demonstrated a significant increase in *ARF* mRNA levels in *SIRT7*-depleted H1299 cells but not in Calu-3 cells (*SI Appendix*, Fig. S1*H*), indicating that SIRT7 not only influences ARF stability but also interferes with gene expression or mRNA stabilization at least in some lung cancer cells. Taken together, we demonstrate that SIRT7 destabilizes ARF by increasing its ubiquitination and subsequent proteasomal degradation while also repressing *ARF* mRNA expression, albeit in a context-dependent manner.

### SIRT7 Directly Interacts with ARF to Prevent Interactions with NPM, Thereby Reducing ARF Stability.

**Fig. 3.**
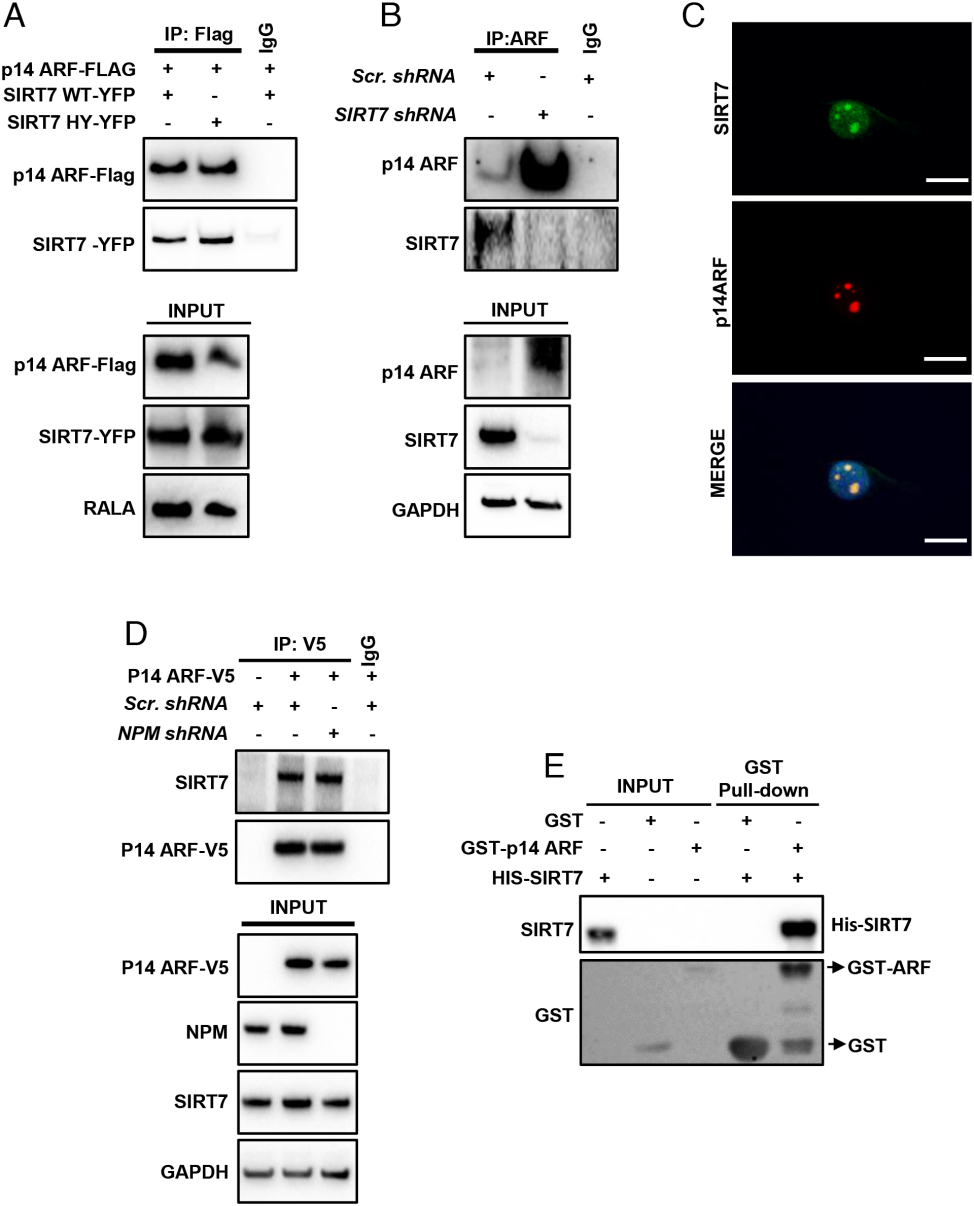
SIRT7 directly interacts with ARF. (*A*) Coupled IP (anti-Flag antibody) and Western blot analysis (anti-Flag and anti-YFP antibody) of 293T HEK cells transfected with YFP-tagged WT and HY mutant SIRT7 in combination with Flag-tagged p14ARF as indicated. Nonimmune immunoglobulin (IgG) was used as negative control. A representative experiment out of three biological replicates is shown. (*B*) Coupled IP (anti-p14ARF antibody) and Western blot analysis (anti-p14 and SIRT7 antibodies) of scrambled and *SIRT7* KD H1299 cells. Nonimmune IgG was used as a negative control. (*C*) IF staining for SIRT7 (green) and p14ARF (red) of H1299 lung cancer cells. Nuclei were counterstained with DAPI. (Scale bar: 20 µm); n = 3. (*D*) Coupled IP (anti-V5 antibody) and Western blot analysis (anti-V5 and SIRT7 antibodies) of stable scrambled and *NPM* KD H1299 lung cancer cell lines transfected with V5-tagged p14ARF as indicated. No difference in binding of exogenous p14ARF with endogenous SIRT7 was observed upon inhibition of *NPM* expression (*Upper*). The inputs of the IP demonstrating efficient depletion of *NPM* are shown in the *Lower*. A representative image of three independent experiments is shown. (*E*) GST-pull down assay of bacterial purified GST-tagged p14ARF and His-tagged SIRT7 demonstrating direct interaction between proteins. A representative image of three independent experiments is shown.

Binding of ARF to NPM is instrumental for the stabilization of ARF protein. Since SIRT7 is a prominent interactor partner of NPM (27, 28), we initially reasoned that binding of SIRT7 to NPM reduces ARF–NPM binding, resulting in destabilization of ARF protein. Coimmunoprecipitation experiments in 293T Human embryonic kidney (HEK) cells revealed that p14ARF forms a complex with SIRT7, which was not influenced by mutation of the catalytic domain in SIRT7 HY mutant (Fig. 3*A*). SIRT7–p14ARF complex formation was corroborated by coimmunoprecipitation of endogenous proteins in H1299 lung cancer cells (Fig. 3*B*). A prerequisite for physiologically relevant protein–protein interactions is the localization of participating proteins in the same subcellular compartment. As expected, IF analysis demonstrated that both SIRT7 and p14ARF localize in the nucleolus in lung cancer cell lines, allowing interactions of the two molecules in this organelle (Fig. 3*C*).

To explore whether SIRT7 requires NPM to interact with ARF, we generated stable NPM KD lung cancer cells overexpressing V5-tagged p14ARF and analyzed binding of exogenous p14ARF with endogenous SIRT7. Surprisingly, depletion of *NPM* did not influence SIRT7 binding to ARF, suggesting a NPM-independent process (Fig. 3*D*). Therefore, we investigated whether SIRT7 is a direct interactor partner of ARF. GST-pull down assays using GST-tagged p14ARF and His-tagged SIRT7 demonstrated that SIRT7 indeed binds directly to ARF (Fig. 3*E*). We concluded that the direct interaction of SIRT7 with ARF destabilizes ARF by preventing its association with NPM.

**Fig. 4.**
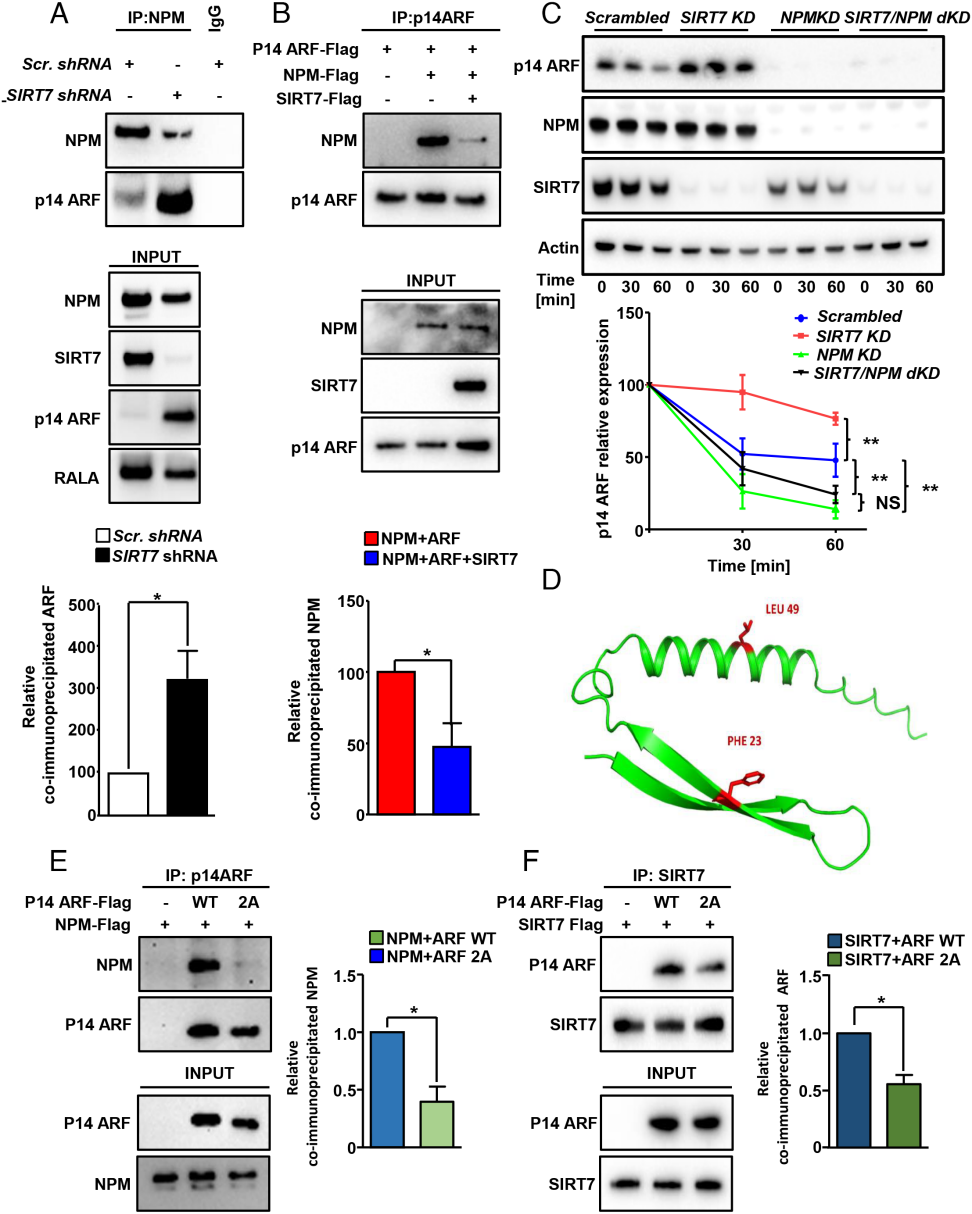
SIRT7 destabilizes ARF by preventing its interaction with NPM. (*A*) Coupled IP (anti-NPM antibody) and Western blot analysis (anti-NPM and p14ARF antibodies) of scrambled and *SIRT7* KD H1299 lung cancer cells (*Upper*). The inputs of the IP are shown in the lower histogram. The relative levels of coimmunoprecipitated ARF normalized on the immunoprecipitated NPM ± SD are quantified in the histograms below (n = 6). (*B*) Coupled IP (anti-ARF antibody) and Western blot analysis (anti-ARF and NPM antibodies) of purified Flag-tagged ARF and NPM incubated in the presence or absence of Flag-tagged SIRT7. A quantification of relative coimmunoprecipitated NPM is shown in the lower histogram (n = 4). (*C*) Western blot analysis of ARF protein levels in stable H1299 cells expressing *SIRT7-* and *NPM-*targeting shRNA alone (KD) or in combination (double KD; dKD) at different time points after treatment with translational inhibitor CHX (50 µg/mL) as indicated. Quantifications of p14ARF levels are given in the graph below (n = 4; two-way Anova statistic test). (*D*) Molecular model of human ARF (residues 1 to 58) suggests that Phe23 and Leu49 (in red) are positioned at the surface of the protein. (*E*) Coupled IP (anti-ARF antibody) and Western blot analysis (anti-Flag antibody) of purified Flag-tagged WT, point mutant ARF (substitution of Phe23 and Leu49 into Alanine; 2A) and Flag-tagged NPM. Quantification of relative coimmunoprecipitated NPM normalized to immunoprecipitated ARF ± SD is shown in the histogram on the *Right* (n = 3). (*F*) Coupled IP (anti-SIRT7 antibody) and Western blot analysis (anti-Flag antibody) of purified Flag-tagged WT, 2A point mutant ARF and Flag-tagged SIRT7. Quantification of relative coimmunoprecipitated ARF normalized to immunoprecipitated SIRT7 ± SD is shown in the histogram on the *Right* (n = 4).

To investigate whether SIRT7 affects the association of NPM with ARF, coimmunoprecipitation experiments were conducted in lung cancer cells after KD of *SIRT7*. We found that depletion of *SIRT7* dramatically increased the binding of ARF to NPM (Fig. 4*A*). To rule out that enhanced coprecipitation of ARF with NPM is caused by increased ARF protein levels in *SIRT7*-depleted cells, we expressed and purified Flag-tagged NPM, ARF, and SIRT7 from separate cultures of 293F SIRT7 KO cells. Purified NPM and ARF were incubated alone or in the presence of SIRT7, followed by IP (immunoprecipitation) using an anti-p14ARF antibody. We found that the presence of SIRT7 significantly reduced the amount of ARF bound to NPM levels, clearly demonstrating that SIRT7 disrupts the binding of ARF to NPM (Fig. 4*B*).

Finally, we wanted to assess whether SIRT7 controls ARF stability in a NPM-dependent manner. ARF protein stability was analyzed in lung cancer cell lines, in which *SIRT7* and *NPM* expression was supressed, either alone or in combination, following treatment with CHX. Concomitant inhibition of NPM prevented enhanced stability of ARF in *SIRT7-*depleted cells. Moreover, no effects on ARF stability were observed when *NPM*-depleted cells were additionally subjected to *SIRT7* KD, conclusively demonstrating that SIRT7 inhibits ARF stability in a NPM-dependent manner (Fig. 4*C*). Analysis of protein stability after CHX treatment of exogenous ARF following coexpression with NPM either alone or in combination with SIRT7 confirmed that SIRT7 reduces NPM-dependent stabilization of ARF (*SI Appendix*, Fig. S1*I*).

To investigate whether SIRT7 and NPM occupy the same binding sites within ARF, we aligned ARF protein sequences from different mammals, which revealed the presence of 2 highly conserved hydrophobic amino acids (Phe 23 and Leu 49). Since Phe 23 and Leu 49 are positioned at the protein surface, a potential involvement in protein–protein interactions seemed likely (Fig. 4*D* and *SI Appendix*, Fig. S2*A*). In agreement with our expectations, mutations of Phe 23 and Leu 49 into alanine significantly reduced SIRT7 and NPM binding to ARF (Fig. 4 *E* and *F*). Moreover, deletion of the N-terminal domain of ARF, where Phe 23 and Leu 49 are located, strongly impaired binding of ARF to NPM and SIRT7, indicating that ARF requires this domain to bind both proteins (*SI Appendix*, Fig. S2 *B*–*D*). However, we found that also the deletion of other domains of ARF disrupted binding to SIRT7 or NPM. We speculate that the deletion of these domains may disturb the proper tridimensional structure of ARF, thereby disabling its interaction with SIRT7 or NPM (*SI Appendix*, Fig. S2 *C* and *D*).

### SIRT7 Stimulates the Expression of Genes Involved in Lung Cancer Progression in an ARF-Dependent Manner.

**Fig. 5.**
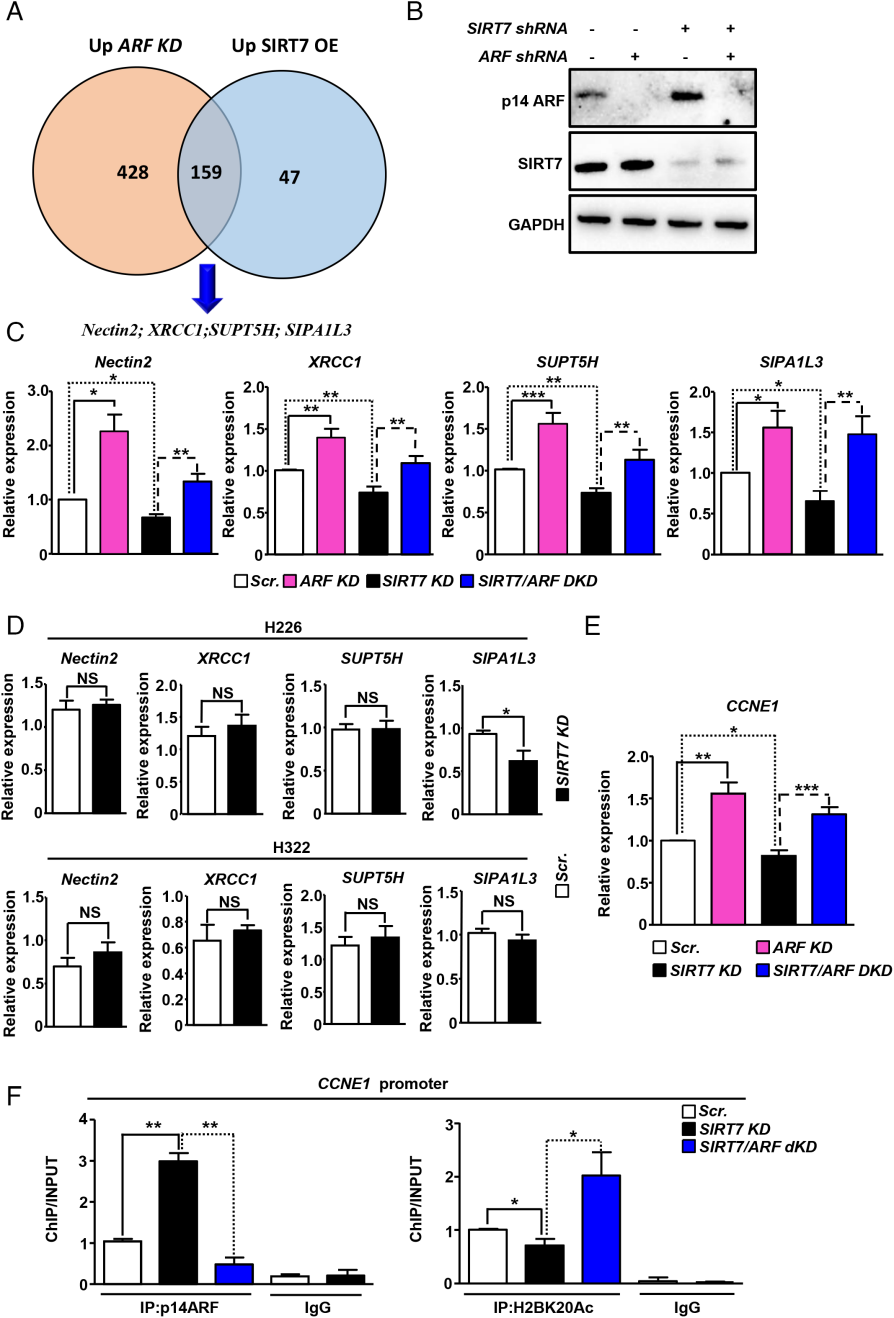
Depletion of *SIRT7* represses expression of genes involved in lung cancer progression in an ARF-dependent manner. (*A*) Venn diagram of significantly up-regulated genes [Log2 fold of change (FC) ≥ 0.58; FDR < 0.05] in *ARF* KD and *SIRT7*–V5 overexpressing H1299 cells as assessed by RNA-sequencing. A total of 159 genes were up-regulated in both datasets including *Nectin2*, *XRCC1*, *SUPT5H,* and *SIPA1L3*. (*B*) Western blot analysis of p14ARF levels in stable scrambled, *SIRT7* KD and *SIRT7*/*p14ARF* dKD H1299 cells. (*C*) RT-qPCR analysis of mRNA expression of indicated genes in cells as in *B*. *GAPDH* was used as loading control (n = 7 for *Nectin2, SUPT5H,* and *SIPA1L3* and n = 9 for *XRCC1*). (*D*) RT-qPCR analysis of mRNA expression in H226 and H322 *ARF*-depleted lung cancer cell lines. *GAPDH* was used as a loading control (n = 4). (*E*) RT-qPCR analysis of mRNA expression of *CCNE1* in stable H1299 cells as in *B*. *GAPDH* was used as loading control (n = 7). (*F*) Chromatin IP analysis of p14ARF (*Left*) and acetylated H2B at lysine 20 (H2BK20; *Right*) enrichment at the promoter of *CCNE1* gene in *SIRT7* KD and *SIRT7/ARF* dKD cells (n = 3).

ARF is a tumor suppressor. Thus, SIRT7-mediated changes in ARF levels may influence expression of genes involved in lung cancer progression. To explore this possibility, we performed RNA-sequencing of H1299 lung cancer cells stably expressing scrambled or *ARF*-targeting *shRNA* (*SI Appendix*, Fig. S3 *A*–*C* and Dataset S1) as well as of H1299 cells expressing V5-tagged SIRT7 (*SI Appendix*, Fig. S3 *D*–*F* and Dataset S2). Five hundred eighty seven genes were up-regulated after KD of ARF, and 206 genes were up-regulated after overexpression of SIRT7. Importantly, 159 of the 206 genes up-regulated due to overexpression of SIRT7 were also up-regulated in cells depleted for *ARF* (Fig. 5*A*). Pathway enrichment analysis revealed that several of the up-regulated genes are involved in lung cancer progression (*SI Appendix*, Fig. S3*G*). Since SIRT7 destabilizes ARF, these data suggest that the effects of SIRT7 on NSCLC are primarily mediated by SIRT7-dependent reduction of ARF levels. Several of the genes concomitantly up-regulated in ARF-depleted and SIRT7-overexpressing cells, such as *Nectin2*, *XRCC1*, *SUPT5H,* and *SIPA1L3,* are critical protumorigenic genes (29–32). To validate that SIRT7 controls expression of these genes in an ARF-dependent manner, we generated stable H1299 cells in which we inhibited *SIRT7* and *ARF* alone or in combination (Fig. 5*B*) and performed RT-qPCR analyses (Fig. 5*C*). We found that depletion of *ARF* up-regulated expression of *Nectin2*, *XRCC1*, *SUPT5H* and *SIPA1L3*, confirming that ARF represses these genes. KD of *SIRT7* reduced expression of these genes, which was prevented by concomitant inhibition of *ARF*, indicating that upregulation of ARF levels in *SIRT7*-deficient cells is responsible for repression *Nectin2*, *XRCC1*, *SUPT5H,* and *SIPA1L3* (Fig. 5*C*).

To further demonstrate that SIRT7 controls expression of these genes involved in tumorigenesis in an ARF-dependent manner, we depleted *SIRT7* in NSCLC cells (H226 and H322) that do not express *ARF* (33) (*SI Appendix*, Fig. S4*A*). Notably, RT-qPCR analysis of *Nectin2*, *XRCC1*, and *SUPT5H* mRNA levels demonstrated that depletion of *SIRT7* had no effects on *Nectin2*, *XRCC1*, and *SUPT5H* expression when *ARF* is absent (Fig. 5*D*). However, depletion of *SIRT7* in *ARF*-negative H226 but not in *ARF*-negative H322 cells suppressed *SIPA1L3* expression, indicating context-dependent effects SIRT7 on *SIPA1L3* that do not depend on ARF (Fig. 5*D*). Remarkably, inhibition of SIRT7 also enhanced the repression of ARF target genes in ARF-positive Calu-3 lung cancer cells (*SI Appendix*, Fig. S4*B*) further supporting the hypothesis that SIRT7 controls expression of *Nectin2*, *XRCC1*, and *SUPT5H* in an ARF-dependent manner.

Previous studies demonstrated that ARF binds to the *CCNE1* promoter to repress expression by recruiting HDAC1 and stimulating HDAC1-mediated deacetylation of H2BK20 (10). Analysis of *CCNE1* mRNA and protein expression in scrambled, *SIRT7* KD and *SIRT7/ARF* double KD H1299 cells revealed that KD of *SIRT7* reduced *CCNE1* expression, which was prevented by inhibition of ARF (Fig. 5*E* and *SI Appendix*, Fig. S4*C*). The same downregulation of *CCNE1* was also apparent in *SIRT7* KD ARF-positive Calu-3 lung cancer cells (*SI Appendix*, Fig. S4*D*) and importantly in lungs of *SIRT7*-mutant mice, demonstrating the physiological relevance of this phenomenon (*SI Appendix*, Fig. S4*E*). In sharp contrast, CCNE1 expression did not change or even increased in ARF-negative *SIRT7*-depleted H226 and H322 lung cancer cells, respectively (*SI Appendix*, Fig. S4*F*). These results indicate that suppression of *CCNE1* following depletion of *SIRT7* occurs in an ARF-dependent manner. Since ARF binds to promoters of target genes and induces epigenetic silencing, we assessed the ARF and H2BK20Ac levels at the *CCNE1* promoter in scrambled, *SIRT7* KD, and *SIRT7/ARF* dKD (double knockdown) cells. Lack of *SIRT7* dramatically increased binding of ARF to the *CCNE1* promoter and reduced H2BK20 acetylation. Correspondingly, inhibition of *ARF* in *SIRT7*-depleted cells prevented the reduction of H2BK20 acetylation (Fig. 5*F*). However, ChIP (Chromatin IP) analysis of H2BK20 acetylation and ARF binding at the promoter of *XRCC1*, *Nectin 2*, *SIPA1L3,* and *SUPT5H* genes revealed the absence of ARF at these gene loci. Since SIRT7 also does not influence H2BK20 acetylation at these regions (*SI Appendix*, Fig. S4 *G* and *H*), we concluded that the SIRT7–ARF axis exerts indirect effects on *Nectin2*, *XRCC1*, *SUPT5H,* and *SIPA1L3*, potentially by regulating transcription factors or other molecules that may control the expression of the aforementioned genes (5). Consistent with the observation that SIRT7 destabilizes ARF regardless of its catalytic activity, expression of either wild-type or catalytically inactive SIRT7 into *SIRT7* knockout lung cancer cells increased expression of genes repressed by ARF (*SI Appendix*, Fig. S5 *A* and *B*). Taken together, these data strongly suggest that SIRT7 controls expression of critical genes involved in lung cancer progression in an ARF-dependent manner.

**Fig. 6.**
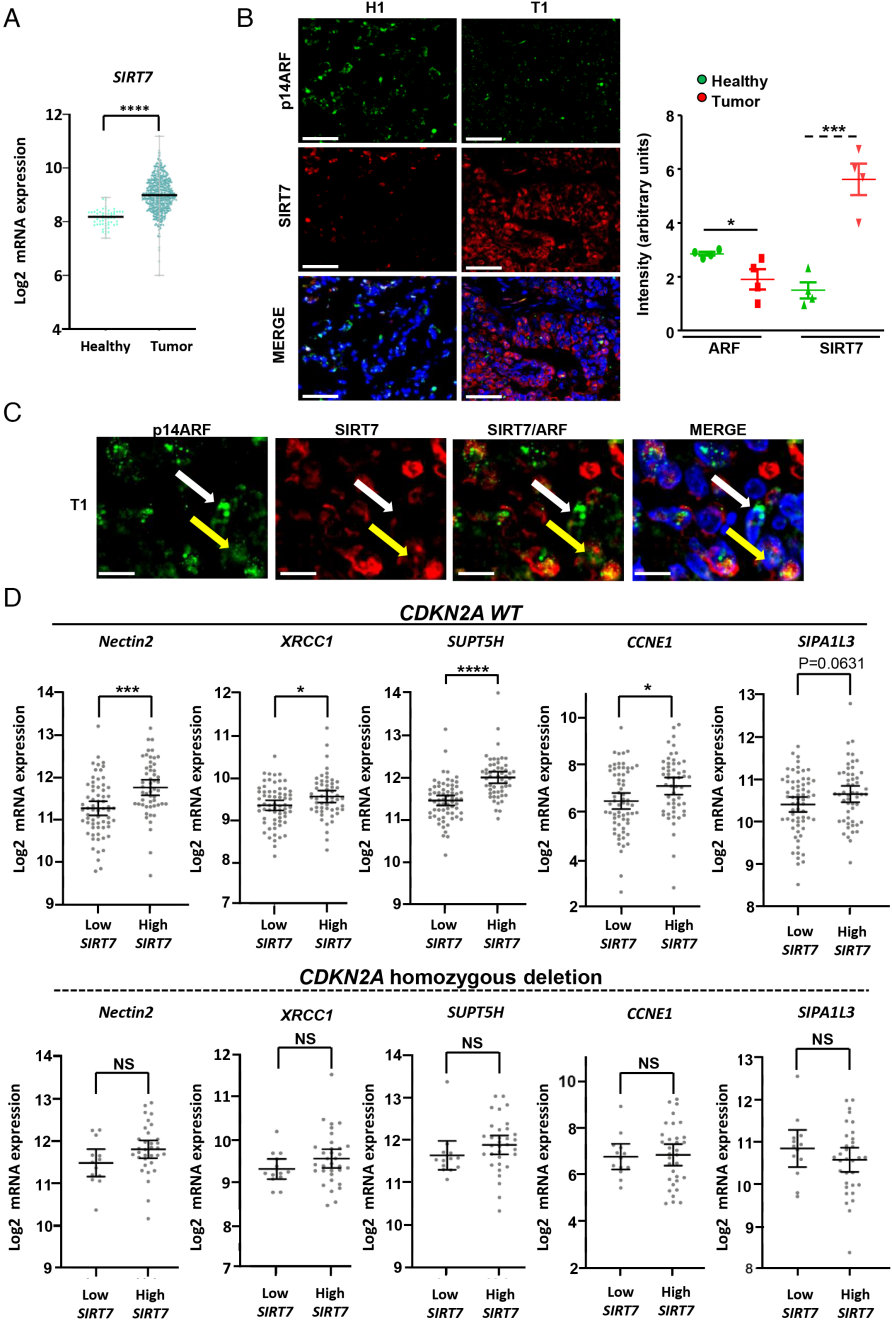
*SIRT7* expression levels inversely correlate with expression of ARF-regulated genes in human lung tumors. (*A*) Scatter plot of Log2 mRNA expression of *SIRT7* in healthy tissues and lung adenocarcinoma samples obtained from the lung adenocarcinoma TCGA study (healthy n = 59; tumor n = 517). (*B*) Representative IF staining of SIRT7 and ARF in healthy human lungs and lung cancers. The histogram on the *Right* shows a quantification of ARF and SIRT7 intensity staining from 4 healthy tissues and 4 lung tumors. (Scale bar: 50 µm.) (*C*) IF staining of SIRT7 and ARF in human lung tumor. Note that individual lung cancer cells displaying high levels of SIRT7 exhibit low levels of ARF in the nucleoli (yellow arrow), and vice versa (white arrow). (Scale bar: 10 µm.) (*D*) Scatter plots of Log2 mRNA expression of indicated genes in lung adenocarcinoma patients with low or high mRNA levels of *SIRT7* harboring WT CDKN2A locus or CDKN2A homozygous deletion (WT CDKN2A locus: low *SIRT7* n = 65; high *SIRT7* n = 53; CDKN2A homozygous deletion low *SIRT7* n = 13; high *SIRT7* n = 33).

Next, we wanted to assess the relevance of the SIRT7–ARF axis in human lung tumors. Bioinformatics analysis of human lung adenocarcinoma patient samples revealed increased expression of *SIRT7* in tumors compared to healthy tissues (Fig. 6*A*). Moreover, we found an inverse correlation between SIRT7 mRNA expression and CDKN2A protein levels in human lung cancers. However, we were unable to distinguish precisely between ARF levels and other molecules encoded by the *CDKN2A* locus, due to annotation problems (*SI Appendix*, Fig. S6*A*). IF analyses of SIRT7 and p14ARF confirmed increased SIRT7 and decreased ARF protein levels in human lung tumors compared to healthy lungs (Fig. 6*B* and *SI Appendix*, Fig. S6*B*). We also observed reduced levels of ARF in individual lung cancer cells expressing high levels of SIRT7 and vice versa (Fig. 6*C* and *SI Appendix*, Fig. S6*C*). Finally, we found that high expression of SIRT7 only correlates with heightened expression of genes normally repressed by ARF in tumors with an intact *CDKN2A* locus but not in those showing deletion of the locus (Fig. 6*D*). These data from human patients imply that inhibition of the SIRT7–ARF axis may restore ARF levels and decrease expression of various genes promoting tumor progression, at least in tumor cells carrying an intact and active *ARF* gene.

### *SIRT7* Depletion Attenuates Proliferation of NSLC Cells in an ARF-Dependent Manner.

**Fig. 7.**
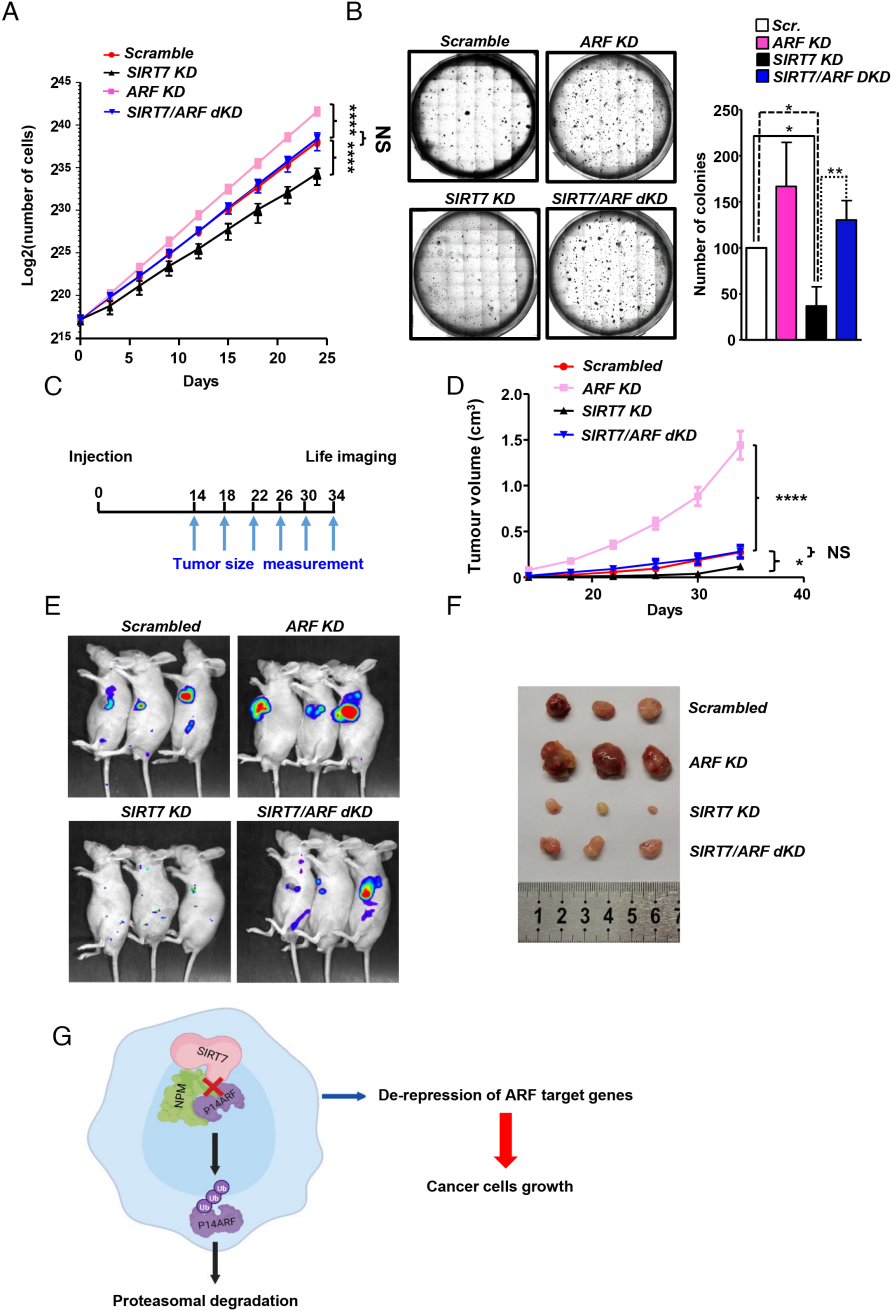
*SIRT7* depletion inhibits lung cancer cells growth in vivo and in vitro in an ARF-dependent manner. (*A*) Growth curves of scrambled, *SIRT7* KD, *ARF* KD, and *SIRT7–ARF* dKD cells. SIRT7 depletion significantly reduces cell proliferation, which is reverted by concomitant inhibition of *ARF* (n = 3; Two-way ANOVA). (*B*) Soft-agar colony formation of scrambled, *SIRT7* KD, *ARF* KD, and *SIRT7/ARF* dKD H1299 cells. The average number of colonies of 3 independent experiments ± SD is shown in the histogram. (*C*) Schematic representation of the subcutaneous mouse xenograft model used in this study. 1 × 10^6^ stable scrambled, *SIRT7* KD, *ARF* KD, and *SIRT7/ARF* dKD cells were injected subcutaneously in 4 to 6-wk-old BALB/c nude mice, and tumor size was measured every 4 d starting from day 14 after injection. (*D*) Fluorescence-based imaging of tumor volumes in the mouse experiment described in *C*. (*E* and *F*) Fluorescence-based imaging (*E*) and macroscopic images of excised tumors (*F*) 34 d after tumor cells injection as described in *C* and *D*. (*G*) Scheme depicting the putative mechanism of SIRT7-mediated ARF regulation in lung cancer cells.

Restored levels of ARF and decreased expression of tumor-promoting genes after loss of *SIRT7* suggested an impact of the SIRT7–NPM–ARF axis on proliferation of lung cancer cells. Thus, we depleted *SIRT7* in H1299 and Calu-3 cells, which strongly reduced the proliferation rate, an effect that was prevented by concomitant inhibition of *ARF* (Fig. 7*A* and *SI Appendix*, Fig. S7 *A* and *B*). Furthermore, *SIRT7*-depleted H1299 cells were less prone to anchorage-independent growth in soft-agar compared to controls (Fig. 7*B*). Depletion of *ARF* in *SIRT7-*deficient cells prevented this phenotype, providing evidence that *SIRT7* depletion retards proliferation of lung cancer cells in an ARF-dependent manner (Fig. 7*B*). Consistent with the dispensability of SIRT7 catalytic activity for inhibition of ARF, we found that expression of either WT or the SIRT7 HY mutant into SIRT7 KO lung cancer cells stimulates proliferation and anchorage-independent growth to a similar extent (*SI Appendix*, Fig. S7 *C* and *D*).

To study the relevance of the SIRT7–ARF axis for lung cancer growth in vivo, we employed a xenograft mouse model. Scrambled, *SIRT7 KD*, *ARF KD,* and *SIRT7/ARF* dKD H1299 cells, labeled by expression of EGFP, were subcutaneously injected into nude mice and tumor growth was monitored by fluorescence imaging every 4 d starting from day 14 after injection (Fig. 7*C*). We found that suppression of *SIRT7* significantly reduced tumor growth, whereas inhibition of *ARF* in transplanted tumor cells reverted this phenotype (Fig. 7 *D*–*F* and *SI Appendix*, Fig. S7 *E* and *F*). Matching our in vitro data, we found that depletion of *SIRT7* reduces expression of *Nectin2*, *XRCC1*, *SUPT5H*, *SIPA1L3,* and *CCNE1* in xenograft-derived tumors, an effect that was reversed by concomitant inhibition of ARF (*SI Appendix*, Fig. S7*G*). The data provide evidence that SIRT7 promotes lung cancer progression by destabilizing ARF independently of its catalytic activity both in vitro and in vivo.

## Discussion

NSCLC is the most common type of lung cancer, which is the leading cause of cancer death, accounting for approximately 1.8 million deaths per year. Eighty five percent of lung cancer patients are diagnosed NSCLC (34). Mutations causing activation of oncogenes or inhibition of tumor suppressors contribute to lung cancer development, but the molecular pathways responsible for initiation and progression of this malignancy remain insufficiently explored. Here, we demonstrate that SIRT7, which is up-regulated in NSCLC, plays an important role in suppression of the tumor suppressor ARF by preventing binding of ARF to NPM.

The tumor suppressor ARF is a critical factor for inhibition of cancer progression. Inactivation of *ARF* due to promoter hypermethylation, homozygous deletions, and mutations is a common event in different malignancies (35). Inactivation of *ARF* in mice accelerates oncogene-induced lung tumorigenesis, increasing tumor size and accumulation of DNA damage (36). Moreover, inhibition of *ARF* occurs in experimental mouse models of carcinogen-induced lung cancer (37) as well as in human patients with NSCLC (38, 39). Several oncogenes inhibit *ARF* to promote tumorigenesis. The oncogenic protein DX2 binds and inhibits ARF thereby preventing oncogene-induced apoptosis and senescence. Other examples include Ataxia telangiectasia mutated (ATM) and CD24. ATM promotes protein phosphatase 1-dependent dephosphorylation of NPM, which disrupts NPM–ARF interactions and promotes ULF-dependent ubiquitination and degradation of ARF (22). The cell surface molecule CD24 promotes prostate cancer progression by destabilizing ARF protein by inhibiting NPM–ARF interactions (19). Since increased expression of CD24 is associated with poorer prognosis in lung cancer, a corresponding role in lung cancer is likely (40). SIRT7 employs a similar strategy as ATM and CD24, initiating ARF degradation by interfering with the interaction of NPM and ARF but employs a proprietary mode of action. SIRT7 does not require its enzymatic deacetylation activity to induce degradation of ARF and does not need to bind to NPM for association with ARF, suggesting that SIRT7 may simply compete with NPM for ARF binding. Since several molecules induce degradation or neutralize ARF, the question arises whether inhibition of individual pathways is sufficient to stabilize ARF. Interestingly, pharmacological inhibition of the DX2–ARF interaction delays tumor growth, suggesting that restoration of ARF in lung cancer can be achieved by manipulation of single pathways, notwithstanding the several factors aiming at its destruction (14). The reason for this surprising phenomenon is currently unclear. Different mechanisms might be employed in different groups of tumors to neutralize ARF or the different pathways preventing ARF–NPM binding interact with each other in a synergistic manner.

Although our study demonstrates that SIRT7-mediated destabilization of ARF does not require its deacetylation activity, we do not want to rule out the potential relevance of other enzymatic reactions catalyzed by SIRT7. SIRT7 also possesses a mono-ADP ribosylation (23) and a desuccinylation (41) activity among others, which may affect the NPM–ARF axis. However, it seems more likely that SIRT7 acts as a competitive inhibitor preventing binding of NPM to ARF. In addition, it may also prevent binding of other molecules. In fact, it has been reported that SIRT7 interacts with ATM, a key regulator of the NPM–ARF pathway in lung cancer cells (22, 42).

SIRT7-dependent destabilization of ARF clearly induced expression of critical genes promoting tumor progression that is normally repressed by ARF, thereby stimulating lung cancer cell proliferation. However, SIRT7 may also abrogate other critical tumor suppressive functions of ARF. ARF reduces cell proliferation by attenuating biogenesis of the ribosomes (6, 7), which is necessary to sustain anabolic processes in highly proliferating cancer cells. SIRT7 is a potent stimulator of ribosome biogenesis (25), which may indicate a dual function of increased SIRT7 expression for stimulating ribosome biogenesis in cancer cells: i) neutralization of the repressive effect of ARF on ribosome biogenesis and ii) direct stimulation of ribosome biogenesis. A similar principle is apparent for the regulation of apoptosis. SIRT7 inhibits apoptosis whereas ARF is potent inducer of apoptosis (4, 43). SIRT7 may exert some of its prosurvival functions in cancer by inhibiting ARF but other processes may contribute. Finally, both ARF and SIRT7 are key regulators of the tumor suppressor p53, although the function of SIRT7 on p53 depends strongly on the context, fluctuating from inhibition to activation (44).

Most of the in vitro studies were performed in H1299 lung cancer cell lines that are defective for p53. Thus, we are certain that the observed effects of SIRT7 on ARF do not depend on p53. Of course, the situation will be different in cancer cells with intact expression of ARF and p53, in which SIRT7 may involve other mechanisms to control the ARF–p53 axis. This needs to be further explored in the future. It seems likely that the oncogenic function of SIRT7 does not exclusively rely on degradation of ARF. SIRT7 was found to repress tumor suppressor genes by epigenetic mechanisms and regulates other critical targets involved in tumorigenesis (26). In fact, our data revealed that SIRT7 not only induces degradation of ARF but also reduces *ARF* mRNA levels, although the effects were much more moderate and cell-type specific. The existence of ARF-independent functions of SIRT7 for controlling tumor cells are also supported by a recent report by Zhao and collaborators (45), who used lung cancer cells that do not express ARF (39).

Given the critical oncogenic role of SIRT7 in different malignancies, specific inhibitors of the enzymatic activity of SIRT7 have been designed as possible candidates for anticancer therapies (46). However, our data demonstrate that the effect of SIRT7 on ARF (and probably other still unexplored targets) does not require deacetylation activity, suggesting that these drugs may only have limited efficiency. Recently, the use of small-molecule-induced protein degradation as in proteolysis-targeting chimeras and hydrophobic tagging technologies has been proposed as an alternative strategy for inhibition of specific oncogenes. Such strategies do not rely on the inhibition of a specific biochemical activity but remove the whole proteins, which theoretically will prevent oncogenic functions of SIRT7 that are not related to its catalytic activity (47).

Altogether, we propose a model in which SIRT7 accelerates lung cancer progression by directly interacting with ARF, preventing the association of ARF with NPM, thus causing ARF ubiquitination and proteasomal-dependent degradation. Essentially, SIRT7 abolishes the capacity of ARF to suppress expression of genes required for proliferation of lung cancer cells, thereby facilitating tumorigenesis (Fig. 7*G*). Pharmacological manipulation of the SIRT7–NPM–p14ARF axis represents an attractive option to target lung cancer cells with an intact and active *ARF* gene.

## Materials and Methods

### Cell Culture and Treatment.

H1299, H226, Calu-3, and PC-14 lung cancer cell lines as well as phoenix-AMPHO cells were purchased from ATCC. H322 cells were purchased from Sigma-Aldrich. 293F SIRT7 KO cells have been described (23). Cells were cultivated in DMEM containing 4.5 g/L glucose supplemented with 10% fetal calf serum (FCS, Sigma-Aldrich), 100 U/mL penicillin, 0.1 mg/mL streptomycin, and 2 mM glutamine (Sigma-Aldrich) at 37 °C in a humidified atmosphere with 5% CO_2_. Calu-3 cells were grown under the same conditions but in medium containing 30% of FCS. Concentrations of inhibitors and duration of treatments for MG132 (Selleckchem) and CHX (Calbiochem) experiments are indicated for each experiment.

### Generation of Stable and KO Cell Lines.

Stable and KO cell lines were generated as described in *SI Appendix*.

### Coimmunoprecipitation and Western Blotting.

Coimmunoprecipitation and Western blot analysis were performed as described (27, 48). Antibodies used in this study are listed in *SI Appendix*, Table S2. For coimmunoprecipitation experiments, 293F SIRT7 KO cells were transfected with either Flag-tagged SIRT7, NPM, or p14ARF. Forty-eight hours posttransfection, cells were harvested and tagged proteins were purified by precipitation with Flag-beads (Sigma-Aldrich) and eluted by incubation with Flag-peptide in RIPA buffer (27). After purification, equal volumes of purified NPM and p14ARF were incubated together in the presence or absence of SIRT7 at 4 °C for 30 min. After incubation, samples were subjected to standard coimmunoprecipitation using anti-p14ARF antibody.

### RNA-Sequencing.

RNA-sequencing analysis was performed as described in *SI Appendix*.

### RNA Extraction and RT-qPCR.

RNA extraction and RT-qPCR were performed as described (27). The sequence of the primers used in this study is provided in *SI Appendix*, Table S3.

### ChIP.

ChIP was performed as described (49) using anti-p14ARF (Santa Cruz Biotech.; Sc-53392) and anti-H2BK20Ac antibodies (Diagenode; C15210010). Immunoprecipitated chromatin was analyzed by qPCR using the primers listed in *SI Appendix*, Table S4.

### Plasmids and Cloning.

Plasmids and cloning details are described in *SI Appendix*.

### IF.

IF experiments were performed as described (27) using anti-p14ARF (Santa Cruz Biotech.; Sc-53392), Anti-Tag (CGY)FP (Evrogen; ab121), or anti-SIRT7 (Cell Signaling Tech.; 5360) antibodies.

### Mice and Xenograft Experiments.

Generation of *Sirt7* knockout mice (50) and xenograft experiments were performed as described (51). Measurement of tumor volume was started on day 14 after injection and was done every 4 d (51). Images of xenografts were acquired using the IVIS Lumina imaging system (Xenogen Corporation, Hopkinto, MA, USA; λex = 488 nm, signal collection: 500 to 700 nm, exposure time = 200 ms) 34 d postinjection. The study was approved by the Ethics Committee of Nankai University (China).

### Analysis of Public Datasets.

Analysis of public datasets was performed as described in *SI Appendix*.

### Statistical Analysis.

Data are expressed as mean ± SD of at least 3 independent biological replicates. Statistical significance was assessed by Student’s *t* test (unless differently specified) using the GraphPad Prism 5.0 Software. *P < 0.05, **P < 0.01, ***P < 0.001, ****P < 0.0001, NS: not significant.

## Supplementary Material

Appendix 01 (PDF)

Dataset S01 (XLSX)

Dataset S02 (XLSX)

## Data Availability

All study data are included in the article and/or supporting information.
