# Supplementary material for: SIRT7 promotes lung cancer progression by destabilizing the tumor suppressor ARF
Source: Proc Natl Acad Sci U S A. 2024 Jun 13;121(25):e2409269121. doi: 10.1073/pnas.2409269121 (PMC11194565; doi:10.1073/pnas.2409269121)
Supplement: Supplementary file 1 — Appendix 01 (PDF) [file pnas.2409269121.sapp.pdf]

PNAS

Supporting Information for
SIRT7 promotes lung cancer progression by destabilizing the tumor suppressor ARF.

Poonam Kumari[1], Shahriar Tarighi[1], Eva Fuchshuber[1], Luhan Li[2], Irene Fernández-Duran[3], Meilin Wang [2], Joshua Ayoson[4], Jose Manuel Castelló-García[3], Andrés Gámez-García[3], Maria Espinosa-Alcantud[3], Krishnamoorthy Sreenivasan[1], Stefan Guenther[1], Mireia Olivella[5,6], Rajkumar Savai[4,7,8], Shijing Yue[2], Alejandro Vaquero[4], Thomas Braun[1,7*], Alessandro Ianni[1,3*]

Corresponding authors:
Alessandro Ianni, **Email:** Alessandro.Ianni@mpi-bn.mpg.de
Thomas Braun, **Email:** Thomas.Braun@mpi-bn.mpg.de

This PDF file includes:

Supporting text
Figures S1 to S7
Tables S1 to S4
Legends for Datasets S1 to S2
SI References

Other supporting materials for this manuscript include the following:

Datasets S1 to S2

1. Supporting Information Text

1.1 Supplementary material and methods

1.1.1 Plasmids and cloning

Generation of SIRT7-YFP and NPM-EGFP plasmids have been described (1). p14ARF-Flag plasmid (Flag-tagged human *ARF* c-DNA cloned into pCMV5 backbone) was a gift from Elizabeth Wilson (Addgene plasmid # 89123). V5-tagged *p14ARF* and *SIRT7* cloned into pLX304 plasmid were purchased from GE Healthcare. For generation of retroviruses, WT and *SIRT7* HY mutant cDNAs were cloned into pMSCV-puro backbones (2). For the GST-pull down assay, *p14ARF* cDNA was amplified from the p14ARF-Flag plasmid using the following primers: Forward: GACGAATTCATGGTGCGCAGG TTCTTGG; Reverse: CTCCTCGAGCTCAGCCAGGTCCACGG and cloned into the PGEX-4T1 vector using EcoRI and XhoI restriction sites. Human-N-terminal His-tagged *SIRT7* was cloned into the pET-30b Vector (novagen) using EcoRI/XhoI restriction enzymes. For cloning of the ARF deletion mutants, ARF cDNA was amplified from the p14ARF-Flag plasmid using the following primers: ΔN (Forward: CGA ATTCAGGAGCCAGCGTC; Reverse: TGGGATCCTCAGCCAGGTCCACG), ΔC mutant (Forward: CGA ATTCGTGCGCAGGTTCTTGG; Reverse CGGGATCCTTAGGGTCGGCGCAGTTG). For ΔM mutant, the N-terminal fragment (amino acids 1-50) and the C-terminal fragment (88-132) were first amplified separately (1-50: Forward: CGAATTCGTGCGCAGGTTCTTGGC and Reverse: AGAGTGGCGGGGCA GTAGCATCAGCA and 88-132: Forward: TGCTGATGCTACTGCCCCGCCACTCT and Reverse: GGG ATCCTCAGCCAGGTCCACGGGCA). Resulting fragments were fused using overlapping PCR (Forward: CGAATTCGTGCGCAGGTTCTTGGC and Reverse: GGGATCCTCAGCCAGGTCCACGGG CA). All mutants were cloned into the pCMV5 plasmid using EcoRI and BamHI restriction sites. ARF point mutants were generated by point mutagenesis as described before (1).

1.1.2 Generation of stable and *SIRT7* KO cell lines

Stable cell lines expressing *SIRT7*- and *ARF*-targeting shRNA or V5-tagged *SIRT7* cDNA were generated as already described (3). The sequences of the shRNA used in this study are listed in **Table S1**. H1299 cells expressing Flag-tagged SIRT7 wild type and SIRT7 catalytic inactive mutant (H187Y;

HY) were generated by employing a retroviral system. Phoenix-AMPHO cells were transfected with 10µg pMSCV plasmid containing SIRT7 WT or SIRT7 HY mutant cDNA together with 1µg of envelope vector (VSV-G/pMD2.G). Medium containing retroviral particles was filtered and polybrene (Sigma-Aldrich) was added to a final concentration of 8µg/mL. Target cells were trypsinized, re-suspended in retroviral particles-containing medium, and plated in new dishes for 48 hours. After incubation, cells were supplemented with fresh medium for additional 24 hours. Retrovirus-infected cells were selected using puromycin (5µg/mL; Sigma-Aldrich) for 48 hours. After selection, cells were grown in medium without puromycin for at least 4 passages before further use.
SIRT7 KO H1299 cells were generated using CRISPR/Cas9 technology as described (4) using the following guide RNA: gRNA 1: CCGCTCCGAGCGCAAAGCGG and gRNA 2: CGAGAGCGCGGACCT GGTAA.

1.1.3 RNA sequencing

RNA was isolated using Direct-zol™ RNA miniprep kit (Zymo Research) following the manufacturer's instructions. To avoid contamination by genomic DNA, samples were treated by on-column DNase digestion (DNase-Free DNase Set, Qiagen). Total RNA and library integrity were verified on LabChip Gx Touch 24 (Perkin Elmer). 1µg of total RNA was used as input for SMARTer Stranded Total RNA Sample Prep Kit - HI Mammalian (Clontech). Sequencing was performed with a NextSeq2000 instrument (Illumina) using v3 chemistry, 1x72bp single end setup.
Trimmomatic version 0.39 was employed to trim reads after a quality drop below a mean of Q20 in a window of 20 nucleotides and keeping only filtered reads longer than 15 nucleotides (5). Reads were aligned versus Ensembl human genome version hg38 (Ensembl release 104) with STAR 2.7.10a (6). Aligned reads were filtered with Picard 2.27.1 to remove duplicates multi-mapping, ribosomal and mitochondrial reads. Gene counts were established with featureCounts 2.0.2 by aggregating reads overlapping exons on the correct strand excluding those overlapping multiple genes as previously described (7). The raw count matrix was normalized with DESeq2 version 1.30.1 (8). Contrasts were created with DESeq2 based on the raw count matrix. Genes were classified as significantly differentially expressed at average count > 5, multiple testing adjusted p-value < 0.05, and -0.585 < log2FC > 0.585. The Ensemble annotation was enriched with UniProt data (Activities at the Universal Protein Resource (UniProt).
All downstream analyses were performed on the normalized gene count matrix. A global clustering heatmap of samples was created based on the euclidean distance of regularized log transformed gene counts. Dimension reduction analyses were performed by principal component analysis (PCA) on regularized log transformed counts using the R packages FactoMineR (9).

1.1.4 Analysis of public datasets

For correlation between relative *SIRT7* mRNA and ARF protein levels in lung cancer cell lines, normalized *SIRT7* gene expression and ARF protein data (ID: Q8N726) of 54 different lung cancer cell lines were obtained from public data available through the Cancer Dependency Portal (https://depmap.org/portal). P-values were obtained after a linear regression fit.
To compare *SIRT7* mRNA expression in healthy tissues and tumour samples, normalized RNA expression data of *SIRT7* in TCGA lung adenocarcinoma data sets were downloaded from the Broad Institute Firehose Pipeline (http://gdac.broadinsti tute.org).
For correlation analysis between *SIRT7* mRNA and CDKN2A protein levels in tumors, gene expression and protein data were obtained from a publicly available dataset of lung adenocarcinoma patients (10) using cBioportal (https://www.cbioportal.org/) for Cancer Genomics (11-13). The analysis was carried using log2 transformed normalized RPKM (reads per kilobase of exon per million reads mapped) values and protein abundance ratios. Correlation analysis was performed using the Spearman's method.
Data for *Nectin2*, *XRCC1*, *SUPT5H*, *SIPA1L3* and *CCNE1* mRNA expression were derived from the TCGA lung adenocarcinoma data set (TCGA, Firehose Legacy) via the cBioPortal (11-13). Patients were grouped according to *SIRT7* expression levels, using the mean SIRT7 mRNA expression and separated into groups harbouring WT *CDKN2A* (threshold -1, +1) or *CDKN2A* homozygous deletion (threshold -0.75, +0.75) based on *CDKN2A* copy number alteration data (CNA) from genomic sequencing of the same tumors.

1.1.5 Human samples

Tumor tissue specimens (T1-T4) as well as adjacent healthy tissues (H1-H4), based on the histopathologic analysis, were collected from patients with non-small cell lung cancer at the time of surgery before chemotherapy at the University Hospital Giessen (Giessen, Germany). H1-T1: female, aged 51; H2-T2 male aged 76; H3-T3 male aged 39; H4-T4 female aged 72. A written informed consent was obtained from all patients.

1.1.6 Protein modelling and multiple sequence alignment.

Since a crystal structure is not available for human ARF or ARF homologues, the AF-Q8N726-F1 model structure from Alphafold database was used (14). The structure was energy minimized using Gromacs5 (15). Pymol (16) was used to generate the corresponding Figure. Multiple sequence alignment of ARF orthologous sequences in mammals was performed using TCoffee (17, 18).

1.1.7 Cell growth assay

Growth curve analysis was performed as described (19)

Supplementary figures and legends

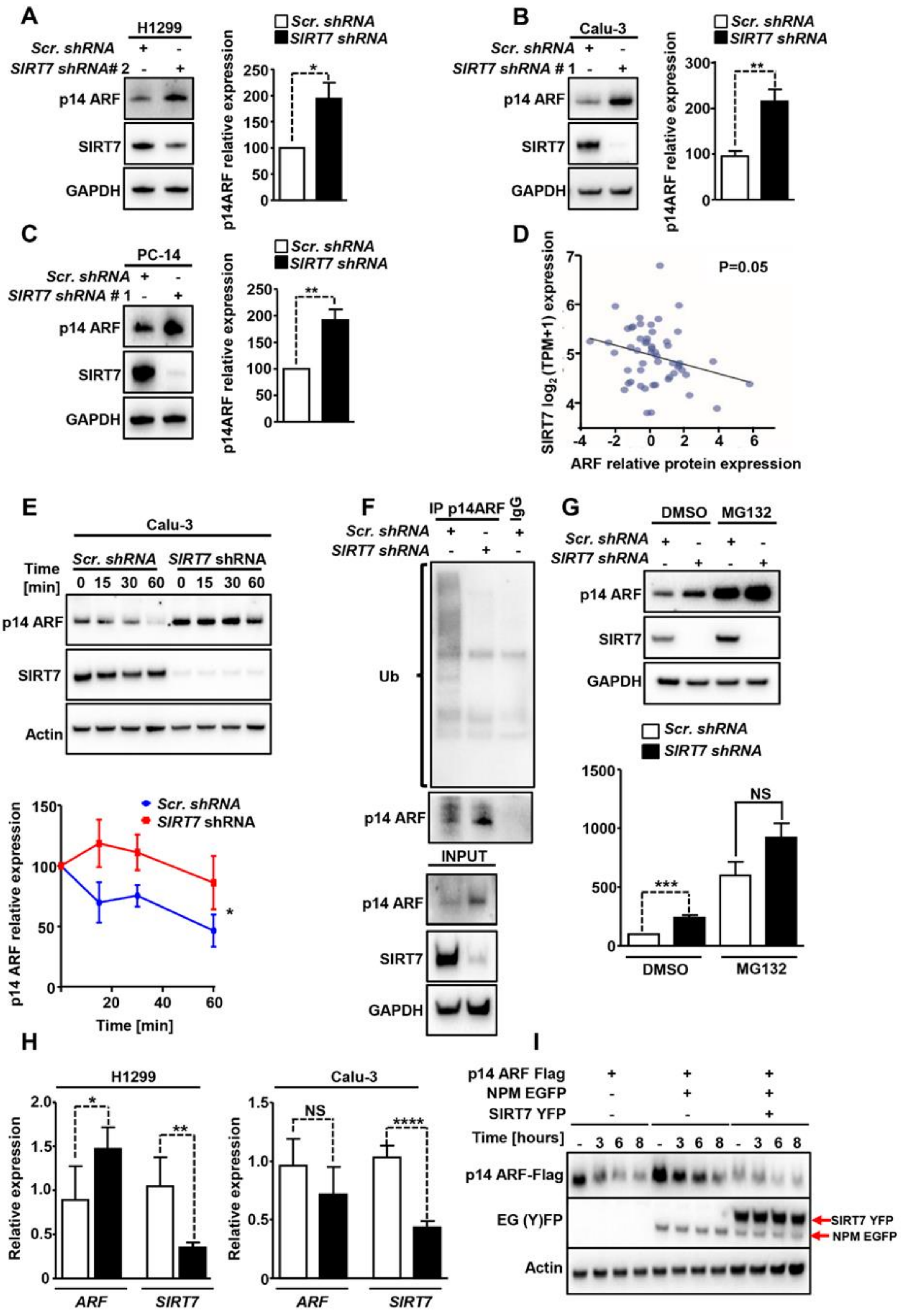

Fig. S1. SIRT7 reduces ARF levels in a NPM-dependent manner. A. Western blot analysis of p14ARF levels in scrambled (*Scr. shRNA*) and *SIRT7* Knockdown (KD; *SIRT7 shRNA # 2*) H1299 lung cancer

cells. Quantification of ARF relative expression ± SD is shown on the right (n=5). **B.** Western blot analysis of p14ARF levels in scrambled (*Scr. shRNA*) and *SIRT7* Knockdown (KD; *SIRT7 shRNA #1*) Calu-3 lung cancer cells. Quantification of relative ARF expression ± SD is shown on the right (n=4). **C.** Western blot analysis of p14ARF levels in scrambled (*Scr. shRNA*) and *SIRT7* Knockdown (KD; *SIRT7 shRNA #1*) PC-14 lung cancer cells. Quantification of relative ARF expression ± SD is shown on the right (n=6). **D.** Plot illustrating the correlation between *SIRT7* mRNA expression and ARF protein levels in various lung cancer cell lines. **E.** Western blot analysis of p14ARF levels in control (scrambled; *Scr. shRNA*) and *SIRT7* KD (*SIRT7 shRNA*) Calu-3 cells at indicated time points after treatment with cycloheximide (CHX; 50µg/mL). Quantifications of p14ARF levels are given in the graph below (n=3; two-way Anova statistic test). **F.** Coupled immunoprecipitation (IP; anti-p14ARF antibody) and Western blot analysis (anti-ubiquitin antibody) of control (scrambled; *Scr. shRNA*) and *SIRT7* KD (*SIRT7 shRNA*) Calu-3 lung cancer cells. A representative blot out of three independent experiments is shown (upper panel). The membrane was re-probed with anti-p14ARF antibody (lower panel). Inputs of the immunoprecipitation are shown below. **G.** Western blot analysis of p14ARF levels in stable scrambled and *SIRT7* KD cells, 5h after treatment with 10µM MG-132. DMSO was used as vehicle. Quantification of ARF levels ± SD is shown in the right histograms (n=7). **H.** RT-qPCR analysis of *p14ARF* and *SIRT7* mRNA expression in control (scrambled; *Scr. shRNA*) and *SIRT7* KD (*SIRT7 shRNA*) H1299 (left panel) and Calu-3 (right panel) lung cancer cells. ß-actin was used as a loading control. Quantification of average mRNA levels relative to ß-actin ± SD is shown in the histograms (H1299: n=5; Calu-3: n=6). **I.** Western blot analysis of Flag-p14ARF levels in 293T HEK transfected with Flag-tagged p14 ARF alone or in combination with EGFP-NPM and YFP-SIRT7 at indicated time points after treatment with cycloheximide (CHX; 50µg/mL).

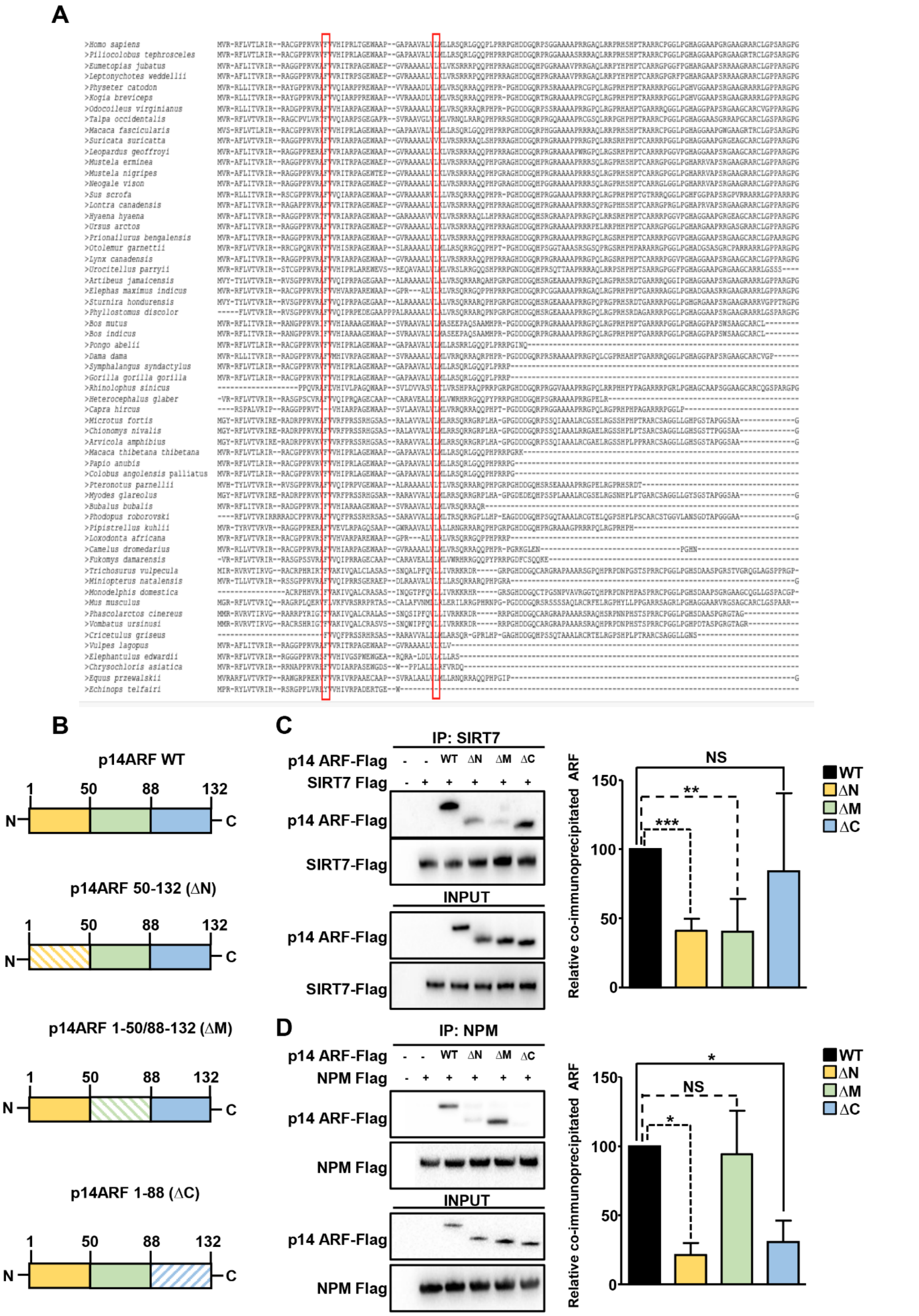

S2. The N-terminal domain of ARF is required for interaction with SIRT7 or NPM. A. Multiple sequence alignment of human ARF orthologous sequences from mammals. Residues Phe23 and Leu49 are highly conserved among mammals (red box). **B.** Schematic representation of ARF deletion mutants used to probe interactions with SIRT7 and ARF. Dashed boxes indicate the deleted regions. The amino

acids contained in the N-terminal ARF mutant (ΔN), middle domain mutant (ΔM) and C-terminal domain mutant (ΔN) are indicated. **C.** Coupled immunoprecipitation (anti-SIRT7 antibody) and Western blot analysis (anti-Flag antibody) of purified Flag-tagged SIRT7 and ARF wild type (WT) or deletion mutants as in B. Quantification of relative co-immunoprecipitated ARF is shown in the right histogram (n=5). **D.** Coupled immunoprecipitation (anti-NPM antibody) and Western blot analysis (anti-Flag antibody) of purified Flag-tagged NPM and ARF WT or deletion mutants as in B. Quantification of relative co-immunoprecipitated ARF is shown in the right histogram (n=4).

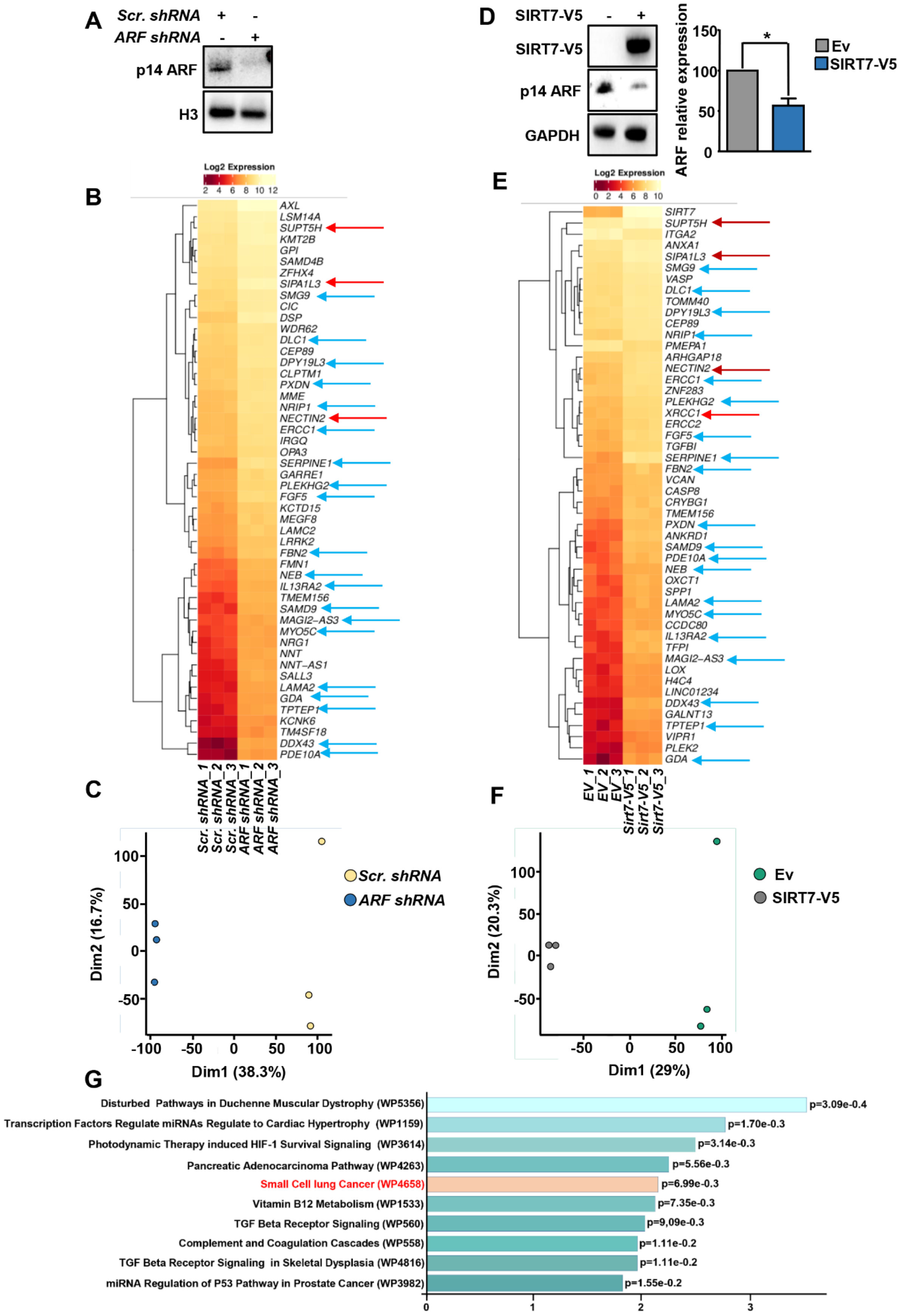

S3. SIRT7 and ARF control expression of genes relevant for proliferation of lung cancer cells. A. Western blot analysis of ARF expression in stable cells lines expressing scrambled or *ARF*-targeting shRNA. Total histone 3 (H3) was used as a loading control. **B.** Heat map of top 50 most significant

differentially expressed genes (DEGs) based on Log2 of expression in scrambled and ARF knockdown H1299 cells (n=3) as assessed by RNA-sequencing (padj; fdr<0,05 Log2FC >+/- 0.585, Mean >5). Arrows indicate genes commonly upregulated in in *ARF* KD and SIRT7-overexpressing H1299 cells. Red arrows indicate genes further validated in this study (see also Fig. S3 E). **C.** Principal component analysis. (PCA) of RNA-sequencing data for samples in **A-B. D.** Western blot analysis of ARF expression in stable H1299 cell lines expressing V5-tagged SIRT7 or an empty vector (Ev). Quantification of four independent experiments is shown on the right histogram. **E.** Heat map of top 50 most significant differentially expressed genes (DEGs) based on Log2 of expression in stable H1299 cells expressing empty vector and V5-tagged SIRT7 as in D. Arrows indicate genes that are also upregulated in *ARF KD* H1299 cells. Red arrows indicate genes further validated in this study (see also Figure S3 B). **F.** PCA of RNA-Sequencing data for samples as in **D-E. G.** GO term analysis of 159 genes that are significantly upregulated both after SIRT7 overexpression and *ARF* knockdown (Fold of change > 0,58) in cells as in **A-C** and **D-F** (see main Fig. 5A). GO term analysis is based on p values, according to the WikiPathway (WP) 2023 human obtained using Enrichr. The top 10 enriched terms for the input gene set are displayed based on the -log10 (p-value). The term at the top has the most significant overlap with the input query gene set. p-values are shown on the right.

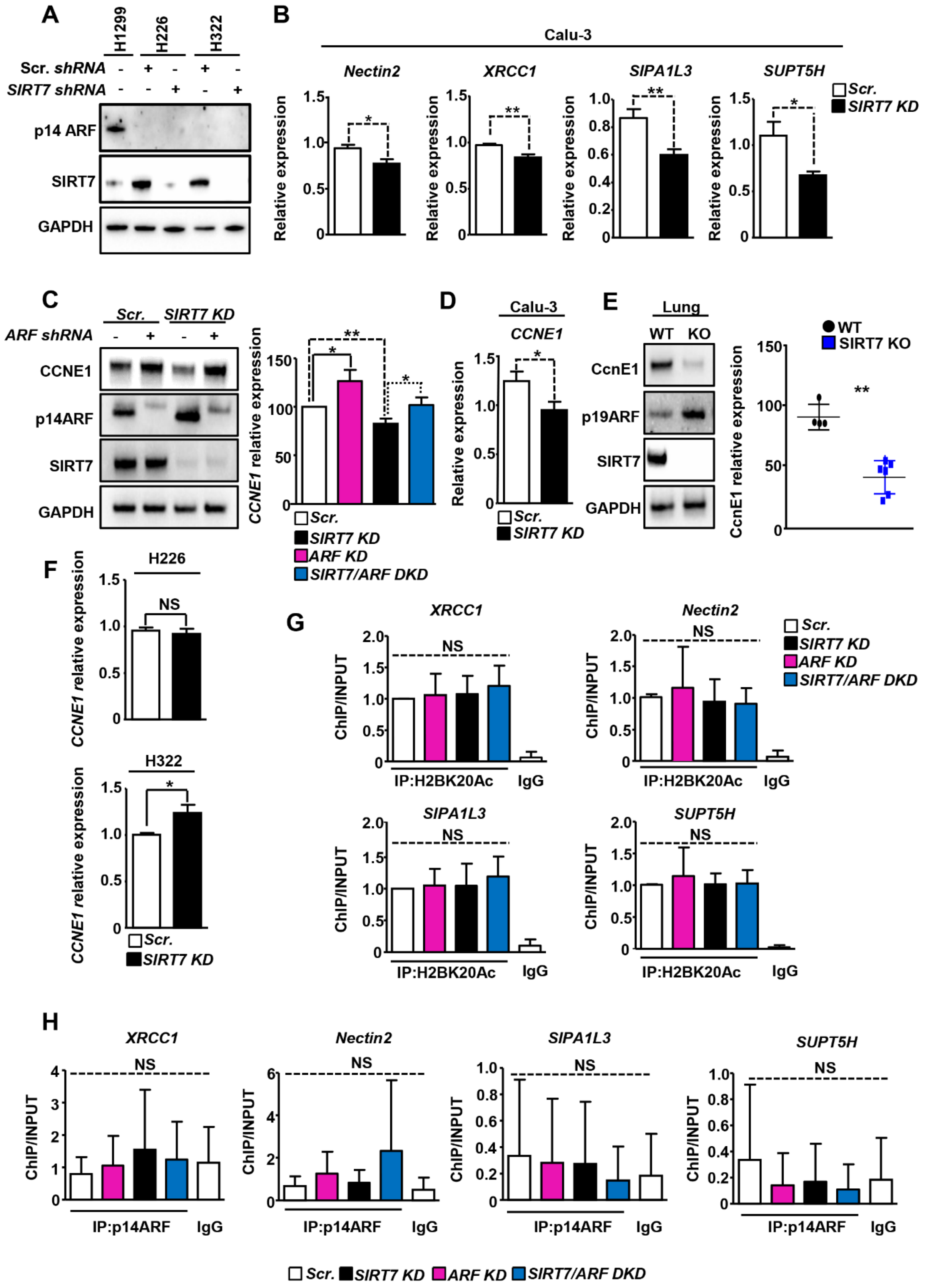

S4. SIRT7 controls expression of a subset of genes in an ARF-dependent manner. A. Western blot analysis of SIRT7 and ARF levels in *ARF*-depleted H226 and H322 lung cancer cells. ARF-expressing H1299 cells were used as a positive control and GAPDH as loading control. **B.** RT-qPCR analysis of

mRNA expression of indicated genes in scrambled (*Scr.)* and *SIRT7* knockdown (KD) Calu-3 lung cancer cells. *β-actin* was used as loading control (n=6). **C.** Western blot analysis of Cyclin E1 (CCNE1) expression in scrambled and *SIRT7* KD H1299 lung cancer cells after shRNA-mediated depletion of *ARF* expression. Quantification of relative CCNE1 levels ±SD is shown in the histogram on the right (n=11). **D.** RT-qPCR analysis of mRNA expression of *CCNE1* in scrambled (Scr.) and SIRT7 knockdown (KD) Calu-3 lung cancer cells. *β-actin* was used as loading control (n=5). **E.** Western blot analysis of lung samples from WT and *SIRT7* KO animals for indicated markers. Quantification of Cyclin E1 (CcnE1) relative levels ±SD is shown in the histogram on the right (n=4 WT and n=6 KO). **F.** RT-qPCR analysis of mRNA expression of *CCNE1* in control (scrambled; *Scr. shRNA*) and *SIRT7* KD (*SIRT7 shRNA***)** ARF-negative H226 and H322 cells. *GAPDH* was used as loading control. Quantification of average mRNA levels ± SD is shown in the histograms (n=4). **G.** Chromatin immunoprecipitation analysis of H2BK20 acetylation at the promoter of indicated genes in *scrambled*, *SIRT7* KD, *ARF* KD and *SIRT7-ARF* double knockdown (*DKD*) H1299 cells (n=5). **H.** Chromatin immunoprecipitation analysis of p14ARF enrichment at the promoter of indicated genes in cells as in G (n=5).

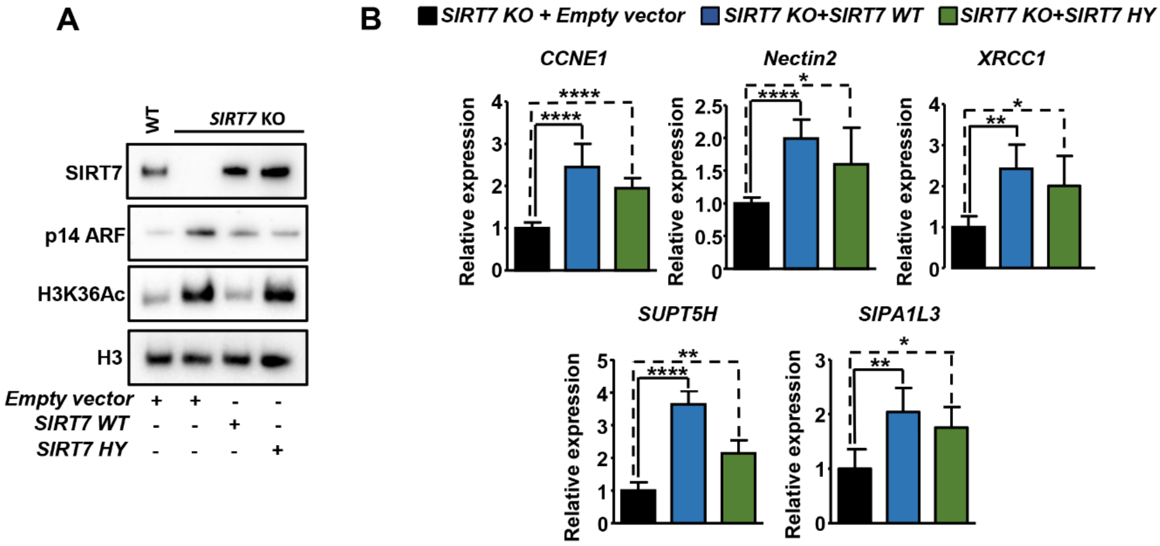

S5. SIRT7 catalytic activity is not required to stimulate expression of genes suppressed by ARF. **A.** Representative Western blot analyses of expression of indicated targets in SIRT7 KO H1299 cells stably expressing exogenously introduced WT or catalytic inactive (HY) SIRT7 at physiological levels. **B.** RT-qPCR analysis of mRNA expression of indicated genes in *SIRT7* knockout H1299 cells lung cancer cells re-expressing WT or the SIRT7 HY mutant as in A. *β-actin* was used as loading (n=5).

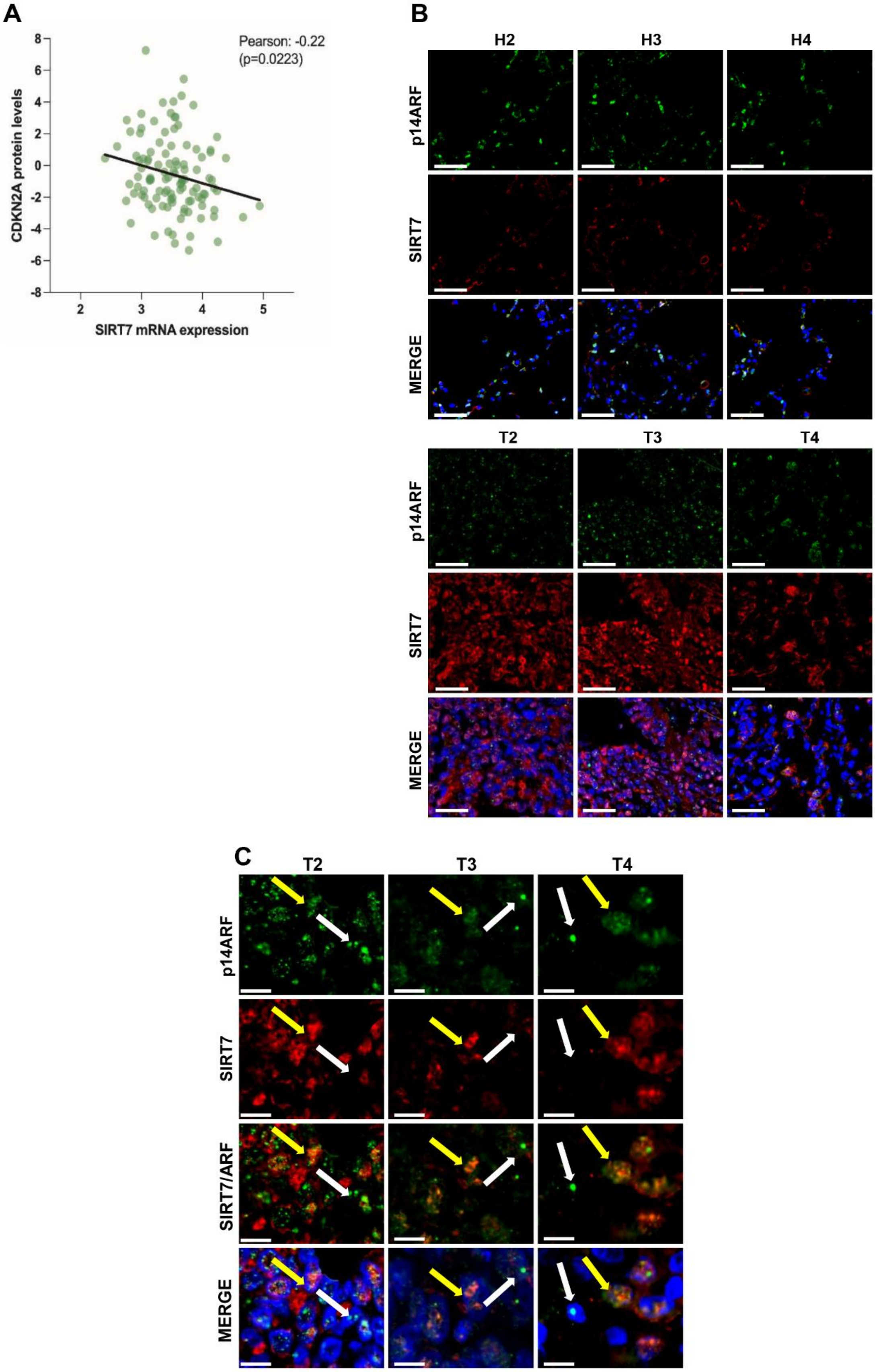

S6. SIRT7 and ARF protein levels inversely correlate in human lung cancers. A. Plot illustrating the inverse correlation between CDKN2A protein and SIRT7 mRNA levels in human lung cancers as assessed by bioinformatics analyses. **B**. Representative immunofluorescence staining of SIRT7 and ARF in healthy human lung tissues and lung cancers. Scale bar 50µm. **C.** Immunofluorescence staining

of SIRT7 and ARF in human lung tumors. Note that individual lung cancer cells displaying high levels of SIRT7 exhibit low levels of ARF in the nucleoli (yellow arrow) and *vice versa* (white arrow). Scale bar 10 µm.

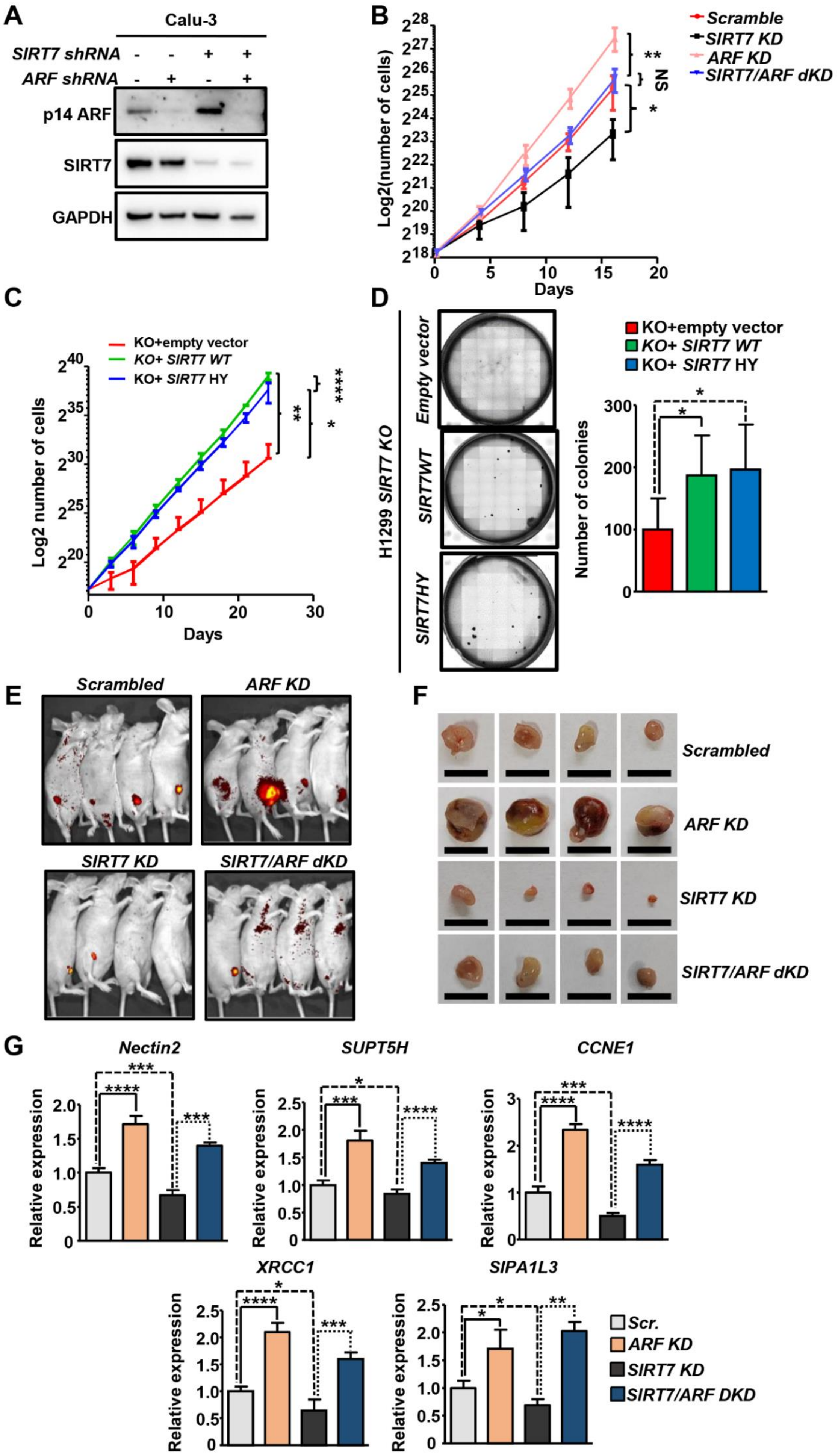
A
Calu-3
SIRT7 shRNA
ARF shRNA
p14 ARF
SIRT7
GAPDH
B
Log2(number of cells)
Days
Scramble
SIRT7 KD
ARF KD
SIRT7/ARF dKD
NS
C
Log2 number of cells
Days
KO+empty vector
KO+ SIRT7 WT
KO+ SIRT7 HY
D
H1299 SIRT7 KO
Empty vector
SIRT7WT
SIRT7HY
Number of colonies
KO+empty vector
KO+ SIRT7 WT
KO+ SIRT7 HY
E
Scrambled
ARF KD
SIRT7 KD
SIRT7/ARF dKD
F
Scrambled
ARF KD
SIRT7 KD
SIRT7/ARF dKD
G
Nectin2
SUPT5H
CCNE1
XRCC1
SIPA1L3
Relative expression
Scr.
ARF KD
SIRT7 KD
SIRT7/ARF DKD

S7. *SIRT7* depletion inhibits lung cancer cells growth *in vitro* and *in vivo* in an ARF-dependent manner. A. Western blot analysis of p14ARF levels in stable scrambled, *SIRT7* KD, *ARF* KD and *SIRT7*/*p14ARF* dKD Calu-3 lung cancer cells. **B.** Growth curves of cells as in A (n=3; Two-way ANOVA). **C.** Growth curves of *SIRT7* KO H1299 cells re-expressing WT or catalytic inactive SIRT7 HY mutant as in Fig. S5A. (n=3; Two-way ANOVA). **D.** Soft-agar colony formation assay of cells as in C. The average number of colonies of 3 independent experiments ± SD is shown in the histogram. **E.** Fluorescence-based imaging of tumor volumes in the mouse xenografts using *scrambled SIRT7* KD, *ARF* KD and *SIRT7/ARF* DKD H1299 cells. **F.** Macroscopic images of excised tumors 34 days after injection of tumor cells in mouse xenografts as in E. **G.** RT-qPCR analysis of mRNA expression of indicated genes in tumors derived from xenografts models (n=4).

Table S1. List of *shRNA* sequences used in this study

Name	Backbone (Source)	Sequence (5´-3´)
Scrambled shRNA#1	plKo.1 (Sigma-Aldrich)	CCTAAGGTTAAGTCGCCCTCGCTCGAGCGAGGGCGACTTAACCTTAGG
SIRT7 shRNA#1	plKo.1 (Sigma-Aldrich)	CCGGGTCCAGCCTGAAGGTTCTAAACTCGAGTTTAGAACCTTCAGGCTGGACTTTTTG
SIRT7 shRNA#2	plKo.1 (Sigma-Aldrich)	CCGGTCCACGGGAACATGTACATTGCTCGAGCAATGTACATGTTCCCGTGGATTTTTG
NPM shRNA	plKo.1 (Sigma-Aldrich)	CCGGCCTAGTTCTGTAGAAGACATTCTCGAGAATGTCTTCTACAGAACTAGGTTTTTG
Scrambled shRNA#2	pGIPZ (Dharmacon)	TGCTGTTGACAGTGAGCGATCTCGCTTGGGCGAGAGTAAGTAGTGAAGCCACAGATGTACTTACTCTCGCCCAAGCGAGAGTGCCTACTGCCTCGGA
p14ARF shRNA	pGIPZ (Dharmacon)	CTTCTAGGAAGCGGCTGCT

Table S2. List of antibodies used for Western blotting, immunoprecipitation and co-immunoprecipitation experiments

Name of antibody	Manufacturer (catalog number)
p14ARF	Santa Cruz Biotech. (Sc-53392)
p19ARF	Abcam (ab227809)
SIRT7	Cell Signaling Tech. (5360)
Anti-Tag(CGY)FP	Evrogen (AB121)
RalA	BD Transduction Laboratories (610221)
Cyclin E1	Cell Signaling Tech. (20808)
Actin	Sigma-Aldrich (A2103)
GAPDH	Cell Signaling Tech. (2118)
NPM/B23	Santa Cruz Biotech. (Sc-56622).
Monoclonal ANTI-FLAG® M2	Sigma-Aldrich (F1804)
Anti-V5 tag	Abcam (ab9116)

Table S3. Sequences of primers used for RT-qPCR

Name	Sequence (5´-3´)
CCNE1 forward	AGCGGTAAGAAGCAGAGCAG
CCNE1 reverse	TTTGATGCCATCCACAGAAA
p14ARF forward	ATGGTGCGCAGGTTCTTGGTGA
p14ARF reverse	GGGGTCGGCGCAGTTGGGCTC
Nectin2 forward	GAGCAGATGGTGTCACGGTCAC
Nectin2 reverse	GAGATGGACACTTCAGGAGGGT
XRCC1 forward	CGGATGAGAACACGGACAGTGA
XRCC1 reverse	GAAGGCTGTGACGTATCGGATG
SUPT5H forward	GGTGTCATCGTGCGACTAGAAC
SUPT5H reverse	CCACAGCAAAGCGGTTGTCCTT
SIPA1L3 forward	CTATGGACGAGGAGACCACATC
SIPA1L3 reverse	GAAGCGTCATGTCCACCGTCTC
GAPDH forward	GAGTCAACGGATTTGGTCGT
GAPDH reverse	GACAAGCTTCCCGTTCTCAG

Table S4. Sequences of primers used for ChIP-qPCR

Name	Sequence (5´-3´)
CCNE1 forward	GCGCAAAGGGGGAAGGGGTA
CCNE1 reverse	GCTCCTTCGCATCCCTGTGGA
Nectin2 forward	AGAACAGGGAGGCTAGAGCG
Nectin2 reverse	CTCGACGGCAGGAGGGCAGC
XRCC1 forward	AGTGGGAGGATCCCTTGG
XRCC1 reverse	ACAGGGTCTTGCTCTCTCA
SUPT5H forward	GGATCTCCTGGGGATGCTTT
SUPT5H reverse	CGACCAGGCTGAAACTCATG
SIPA1L3 forward	CAATTTAGGCCGGGGTCTG
SIPA1L3 reverse	TTTCTCCTTTCTCCTGGCCC

Legends for Datasets

Dataset 1: Raw data of the RNA sequencing analyses performed in H1299 lung cancer cells expressing scrambled or *ARF*-targeting *shRNA*.

Dataset 2: Raw data of the RNA sequencing analyses performed in H1299 lung cancer cells expressing empty vector or V5-tagged *SIRT7*.

SI References

1. A. Ianni *et al.*, SIRT7-dependent deacetylation of NPM promotes p53 stabilization following UV-induced genotoxic stress. *Proc Natl Acad Sci U S A* **118** (2021).
2. N. G. Simonet *et al.*, SirT7 auto-ADP-ribosylation regulates glucose starvation response through mH2A1. *Sci Adv* **6**, eaaz2590 (2020).
3. A. Ianni, S. Hoelper, M. Krueger, T. Braun, E. Bober, Sirt7 stabilizes rDNA heterochromatin through recruitment of DNMT1 and Sirt1. *Biochem Biophys Res Commun* **492**, 434-440 (2017).
4. F. A. Ran *et al.*, Genome engineering using the CRISPR-Cas9 system. *Nat Protoc* **8**, 2281-2308 (2013).
5. A. M. Bolger, M. Lohse, B. Usadel, Trimmomatic: a flexible trimmer for Illumina sequence data. *Bioinformatics* **30**, 2114-2120 (2014).
6. A. Dobin *et al.*, STAR: ultrafast universal RNA-seq aligner. *Bioinformatics* **29**, 15-21 (2013).
7. Y. Liao, G. K. Smyth, W. Shi, featureCounts: an efficient general purpose program for assigning sequence reads to genomic features. *Bioinformatics* **30**, 923-930 (2014).
8. M. I. Love, W. Huber, S. Anders, Moderated estimation of fold change and dispersion for RNA-seq data with DESeq2. *Genome Biol* **15**, 550 (2014).
9. S. Le, J. Josse, F. Husson, FactoMineR: An R package for multivariate analysis. *Journal of Statistical Software* **25**, 1-18 (2008).
10. M. A. Gillette *et al.*, Proteogenomic Characterization Reveals Therapeutic Vulnerabilities in Lung Adenocarcinoma. *Cell* **182**, 200-225 e235 (2020).
11. E. Cerami *et al.*, The cBio cancer genomics portal: an open platform for exploring multidimensional cancer genomics data. *Cancer Discov* **2**, 401-404 (2012).
12. J. Gao *et al.*, Integrative analysis of complex cancer genomics and clinical profiles using the cBioPortal. *Sci Signal* **6**, pl1 (2013).
13. I. de Bruijn *et al.*, Analysis and Visualization of Longitudinal Genomic and Clinical Data from the AACR Project GENIE Biopharma Collaborative in cBioPortal. *Cancer Res* **83**, 3861-3867 (2023).
14. J. Jumper *et al.*, Highly accurate protein structure prediction with AlphaFold. *Nature* **596**, 583-589 (2021).
15. S. Páll, M. J. Abraham, C. Kutzner, B. Hess, E. Lindahl, Tackling Exascale Software Challenges in Molecular Dynamics Simulations with GROMACS. *Lect Notes Comput Sc* **8759**, 3-27 (2015).
16. W. L. DeLano, Use of PYMOL as a communications tool for molecular science. *Abstr Pap Am Chem S* **228**, U313-U314 (2004).
17. H. M. Berman *et al.*, The Protein Data Bank. *Nucleic Acids Res* **28**, 235-242 (2000).
18. C. Notredame, D. G. Higgins, J. Heringa, T-Coffee: A novel method for fast and accurate multiple sequence alignment. *J Mol Biol* **302**, 205-217 (2000).
19. M. Liontos *et al.*, Deregulated overexpression of hCdt1 and hCdc6 promotes malignant behavior. *Cancer Res* **67**, 10899-10909 (2007).
